# Genome-Wide Identification of the *Castanea mollissima* MADS-Box Gene Family and Its Potential Roles in Female Flower Development Based on Transcriptomic and Morphological Analyses

**DOI:** 10.3390/ijms27208994

**Published:** 2026-10-10

**Authors:** Liangyao Zeng, Xiaoyan Hu, Hao Yang, Jia Song, Ruxin Guo, Haie Zhang, Jingzheng Zhang, Chunlei Guo

**Affiliations:** 1 Engineering Research Center of Chestnut Industry Technology, Ministry of Education, Hebei Normal University of Science and Technology, Qinhuangdao 066000, China; 15884448991@163.com (L.Z.); y3160125981@163.com (H.Y.); m18047148606_1@163.com (J.S.); 18231468069@163.com (R.G.); zhang33haie4@163.com (H.Z.); zhangjingzheng@126.com (J.Z.); 2 Hebei Key Laboratory of Horticultural Germplasm Excavation and Innovative Utilization, Qinhuangdao 066000, China; 3 Hebei Higher Institution Application Technology Research and Development Center of Horticultural Plant Biological Breeding, Qinhuangdao 066000, China; 4 College of Animal Science and Technology, Hebei Normal University of Science and Technology, Qinhuangdao 066000, China; 13001423758@163.com

**Keywords:** *Castanea mollissima*, more female cultivars, female flower development, transcriptome sequencing, gene family identification, gene-family evolution

## Abstract

*Castanea mollissima* is an important woody food crop in China. Late, sparse, and unstable female flower differentiation constrains the development of the chestnut industry. Here, two more-female cultivars, ‘Longquanzhuang No. 2’ (LQ) and ‘Yanqiu’ (YQ), and the control cultivar ‘Yanshanzaofeng’ (ZF) were sampled at five stages after budbreak (7–35 d) for morphological observation and transcriptome sequencing. Morphological divergence became evident at 21 d after budbreak. Transcriptome analysis identified 2223 differentially expressed genes specific to the LQ–ZF comparison at 21 d and 791 inter-cultivar differentially expressed genes shared across 21–35 d. GO enrichment analysis showed significant enrichment in transcription factor activity and related pathways. Transcription factor prediction identified *MADS-box* genes as candidates, and 60 *MADS-box* members were identified in Chinese chestnut. These comprised 29 Type I members and 31 Type II members, with the latter classified into 13 subfamilies. Integrated analysis of gene-expression patterns and predicted protein interactions showed that more Type II genes were differentially expressed in female flowers and that expression patterns differed between the more-female cultivars and the control. The contrasting expression patterns of putative positive and negative CmMADS regulators may be associated with differences in female flower developmental capacity among these cultivars. RT-qPCR analysis of four representative *CmMADS* genes showed that genes expressed at higher levels in female flowers generally increased during development, consistent with the transcriptome data. These findings reveal morphological divergence and dynamic *CmMADS* expression during female flower development in two more-female chestnut cultivars and a control cultivar, providing a basis for investigating the underlying molecular regulatory network, but functional validation is currently limited to transcriptome-level prediction, requiring further experimental verification.

## 1. Introduction

The chestnut (*Castanea mollissima* Blume) belongs to the genus Castanea in the family Fagaceae. Yanshan chestnut is a renowned geographical indication product in China and is valued for its high quality. Despite the development of mature cultivation practices, excessive male flowers, insufficient female flowers, and low female flower differentiation rates remain unresolved. These constraints reduce yield per unit area and economic returns, thereby limiting the sustainable development of the chestnut industry [1]. After budbreak, female flowers enter morphological differentiation, which often occurs concurrently with physiological differentiation. The differentiation period is short and concentrated, lasting approximately 60 d [2]. Chestnut female flowers contain stamens positioned below the pistil stigma [3], suggesting that the floral primordia retain the potential for hermaphroditic development. Inflorescence type and sex differentiation may therefore be regulated during floral-bud differentiation through molecular, physiological, and cultivation interventions [4]. Female flower differentiation also varies among cultivars. Long-term field surveys and germplasm collection identified LQ and YQ as more-female cultivars, whereas ZF was selected as a conventional control. LQ and YQ produced 3.2 and 3.3 female flower clusters per fruiting shoot, respectively, compared with 1.6 in ZF. The corresponding ratios of female flower clusters to male inflorescences were 1:2.4, 1:4.3, and 1:5.9 [2]. Cytological observations indicated broadly similar developmental schedules following budbreak in mid-April. However, LQ and YQ showed more advanced differentiation and structures more conducive to female flower-cluster development when mixed inflorescences became visible. Previous studies have examined floral anatomy [5,6,7], trait variation, endogenous hormones [2], and floral-bud transcriptomes in individual cultivars [8]. Systematic comparisons among cultivars with contrasting capacities to produce more female flowers remain limited, particularly for more-female germplasm. Elucidating the regulation of chestnut female flower development is therefore important for understanding the molecular basis of yield formation and improving female flower differentiation through molecular breeding.

Transcription factors regulate target-gene expression in diverse biological processes [9]. Floral organ identity is classically described by the ABC model [10], which was later expanded to include D- and E-class functions [11,12]. Most ABCDE genes belong to the MIKC-type MADS-box transcription factor family. *MADS-box* transcription factors have been studied extensively in Arabidopsis floral organ development. Based on their structural and evolutionary characteristics, *MADS-box* genes are classified into Type I SRF-like (serum response factor-like) and Type II MEF2-like (myocyte enhancer factor 2-like) genes [13]. Type II genes have more complex structures and are further divided into the MIKC^C^ and MIKC* groups [14]. MIKC* genes generally function in male and female gametophyte development [13,15], whereas MIKC^C^ genes have broader roles in floral organ development [16,17]. The MIKC^C^ group contains at least 14 subfamilies, including STK (SEEDSTICK), PI (PISTILLATA), AGL12 (AGAMOUS-LIKE 12), and SEP (SEPALLATA) [18,19]. In the ABCDE model, MADS-box proteins form transcriptional complexes that specify sepals (A), petals (A+B+E), stamens (B+C+E), carpels or pistils (C+E), and ovules (D) [18,19,20]. AP1 and AP2 are A-class genes; PI and AP3 are B-class genes; AG is a C-class gene; STK/AGL11 and SHP are D-class genes; and SEP1–SEP4 are E-class genes [21,22,23,24,25]. Type II *MADS-box* genes also contribute to seed development [25,26]. SEP genes, including TM29 in tomato and FaMADS9 in strawberry, regulate floral organ development, meristem identity, and fruit development and ripening [27,28]. STK promotes coordination between seed and fruit development in Arabidopsis [25], whereas AP1, SHP1, and SHP2 jointly regulate valve separation and seed dispersal [29]. Genome-wide identification and functional characterization of MADS-box families have been conducted in many organisms [30,31], but their roles in chestnut remain incompletely understood.

Genome-wide surveys provide a foundation for investigating gene-family function [19,32]. MADS-box families contain 107 members in Arabidopsis, 105 in poplar, 32 in rice, and 131 in tomato [33,34,35,36]. These transcription factors are central to floral organ identity [37]. For example, *pMADS4* and *pMADS12* regulate floral development in petunia [38], whereas the tomato AG-subfamily genes TAG1 and TAGL1 are expressed primarily in stamens and carpels [39]. Mutations in B- or C-class genes, including *OsMADS3*, *BcAP3*, and *PbTM6a*, disrupt anther development and can cause male sterility [40,41,42]. In *Eucommia ulmoides*, co-expression of two B-class genes increases stamen number, whereas overexpression of an A-class gene reduces it [43]. In chestnut, Qiu et al. [44] identified *MADS-box* genes associated with male and female flower development. Liu et al. conducted a genome-wide survey and characterized their expression across floral organs. Cheng et al. subsequently showed that *CmAP1* promotes flowering and contributes to petal development. However, these studies mainly examined individual cultivars and did not resolve dynamic *MADS-box* expression among cultivars with contrasting female flower phenotypes.

To identify genes potentially involved in chestnut female flower development, we compared the more-female cultivars LQ and YQ with the control cultivar ZF. Morphological changes and transcriptomes were examined at key developmental stages, and comparative DEG analysis identified *MADS-box* transcription factors as candidates. Genome-wide analysis detected 60 *CmMADS* genes, classified by the presence of a K-box domain into 29 Type I and 31 Type II genes. Four representative genes were validated by RT-qPCR. *CmMADS8*, *CmMADS36*, and *CmMADS56* were highly expressed in the more-female cultivars and generally increased during development, whereas *CmMADS16* showed the opposite pattern. These expression profiles reveal dynamic differences between cultivars producing more female flowers and the control and identify candidate *CmMADS* genes for functional investigation. The multi-cultivar time-series analysis helped distinguish expression patterns from cultivar-specific variation and strengthened the association between candidate genes and female flower development.

## 2. Results

### 2.1. Morphological Observations on the Early Development of Chestnut Mixed Inflorescences and Female Flowers

**Figure 1:**
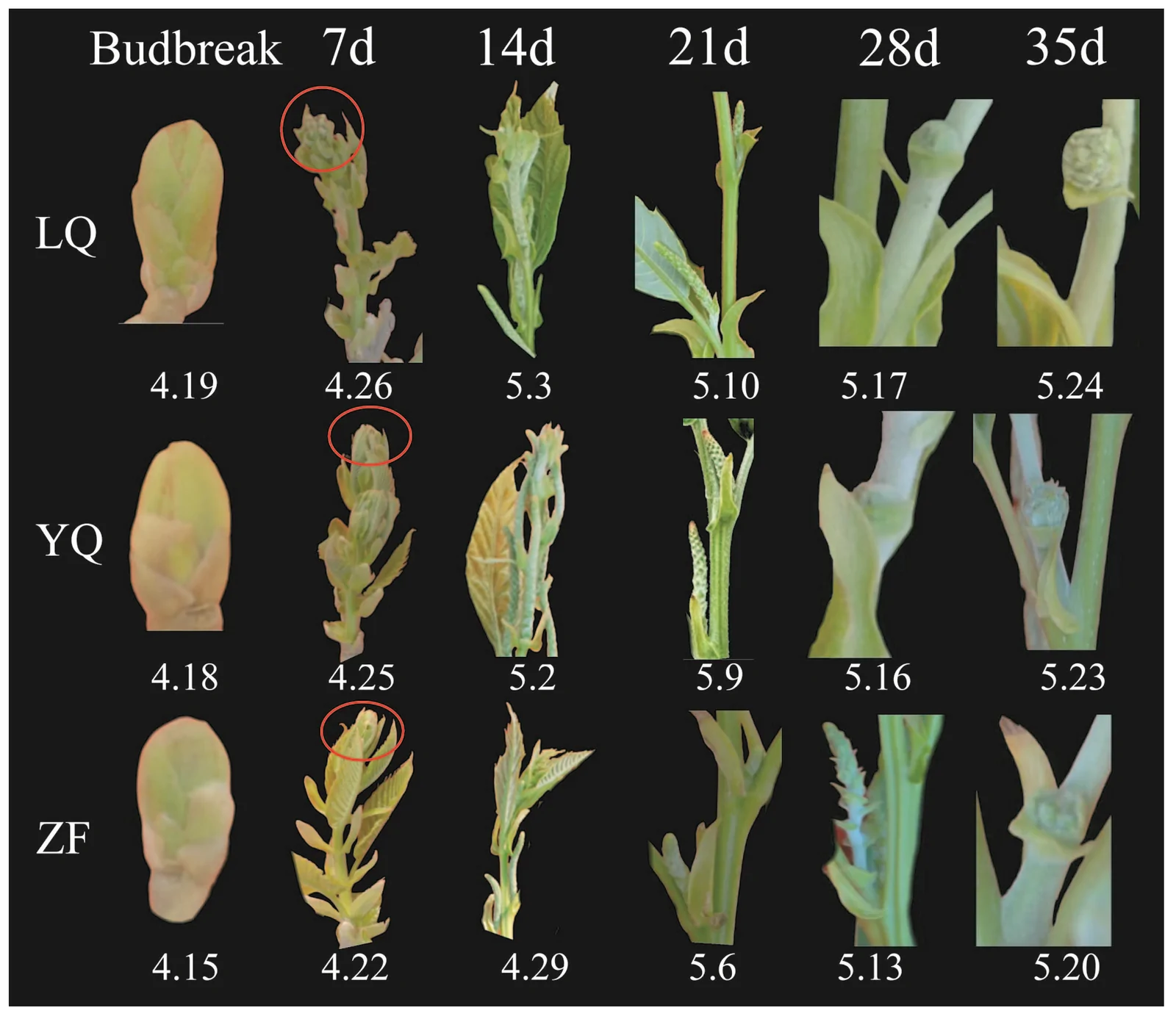
Morphological development of mixed inflorescences and female flowers in LQ, YQ, and the control ZF. LQ, ‘Longquanzhuang No. 2’; YQ, ‘Yanqiu’; ZF, ‘Yanshanzaofeng’. Figure numbers: Germination days and Sampling time. Red circle: 7d sampling region.

To identify critical stages of chestnut female flower development, we compared inflorescence morphology in LQ, YQ, and ZF at 7, 14, 21, 28, and 35 d after budbreak (Figure 1).

At 7 d after budbreak, during inflorescence primordium differentiation, female and male flowers could not be distinguished visually, indicating an early stage of sex differentiation. By day 14 after budbreak, the trees had entered the leaf-unfolding stage and the inflorescences had begun to elongate; however, female and male floral structures remained morphologically indistinguishable. By day 21, sepal primordia differentiation began, and ZF mixed inflorescences developed more slowly than those of the two more-female cultivars, LQ and YQ. This divergence suggests that cultivar-specific morphological differentiation became evident at this stage. At 28 d after budbreak, pistil primordia differentiation was underway and hermaphroditic inflorescences became visible. Previous cytological observations showed that the central floral primordia were more differentiated in LQ and YQ than in ZF [2]. Female flower clusters were also more clearly visible in LQ and YQ, indicating that early female flower development was essentially complete. By day 35 after budbreak, all female flower clusters were clearly distinguishable, and those of LQ and YQ had become visible earlier than those of ZF.

Phenotypic observations showed that chestnut female flower development was dynamic, with the greatest divergence emerging at approximately 21 d after budbreak. Compared with ZF, morphological differentiation began earlier in the LQ and YQ, which had largely completed early differentiation by 28 d. In contrast, female flower development in ZF may have been delayed or impaired at this stage. These morphological differences guided the selection of samples for transcriptome sequencing and subsequent investigation of the underlying molecular mechanisms (Table S1).

### 2.2. Quality Assessment of Transcriptome Sequencing Data

Pearson’s correlation coefficient was used to assess agreement among biological replicates. Values of R approaching 1 indicate strong correlations between replicate samples (Figure S1A). Biological replicates from the same developmental stage were highly correlated, confirming their suitability for downstream analysis. In the principal component analysis (PCA; Figure S1B), each group of three points formed a compact cluster representing the three biological replicates of one sample. This clustering further supported the reproducibility and reliability of the transcriptome data.

Sequencing yielded 441.4 Gb of clean bases, with at least 7 Gb per sample. The error rate was 0.01%, GC content ranged from 43.35% to 44.37%, and Q20 exceeded 98% (Table S2). Across the 45 quality-controlled samples, 89.24–90.54% of clean reads mapped to the chestnut genome, and more than 77% mapped to exons (Table S3). These metrics confirmed that the sequencing data were suitable for downstream bioinformatic analyses. The complete DEG lists and processed expression matrices are provided in Table S8.

### 2.3. Analysis of Differentially Expressed Genes Among Cultivars

We compared LQ and YQ with ZF at each developmental stage. The LQ7d vs. ZF7d, LQ14d vs. ZF14d, LQ21d vs. ZF21d, LQ28d vs. ZF28d, and LQ35d vs. ZF35d comparisons identified 4140, 3544, 8113, 6698, and 6339 DEGs, respectively (Figure S2A). The corresponding YQ vs. ZF comparisons identified 4204, 4507, 3609, 3597, and 6893 DEGs (Figure S2B). At 21 d, when sepal primordia differentiation began, the number of DEGs specific to LQ vs. ZF increased sharply. We identified 2223, 1039, and 1425 DEGs unique to the LQ vs. ZF comparison at 21, 28, and 35 d, respectively. This increase coincided with the differentiation of mixed inflorescences and the morphological divergence between LQ and ZF at 21 d. By 35 d, all female flower clusters were clearly distinguishable, and both more-female cultivars showed their greatest DEG numbers relative to ZF. Although both LQ and YQ produced more female flowers, their DEG profiles differed. The list of DEGs used for analysis is provided in Table S8.

We used 7 d as the baseline for dynamic comparisons because it represented an early stage of female flower morphogenesis. Comparisons of LQ at 14, 21, 28, and 35 d with LQ at 7 d identified 1764, 8197, 6908, and 5618 DEGs, respectively. The corresponding comparisons in YQ identified 3633, 2803, 4338, and 6246 DEGs, whereas those in ZF identified 1169, 4545, 4019, and 6882 DEGs. Upregulated and downregulated DEG counts are provided in Table S4. The number of DEGs in LQ also increased sharply at 21 d relative to 7 d, and upregulated genes outnumbered downregulated genes in both more -female chestnut cultivars. These results associate 21 d after budbreak with a key transition in female flower development, potentially corresponding to the emergence of hermaphroditic inflorescences.

### 2.4. GO Enrichment Analysis of Differentially Expressed Genes

**Figure 2:**
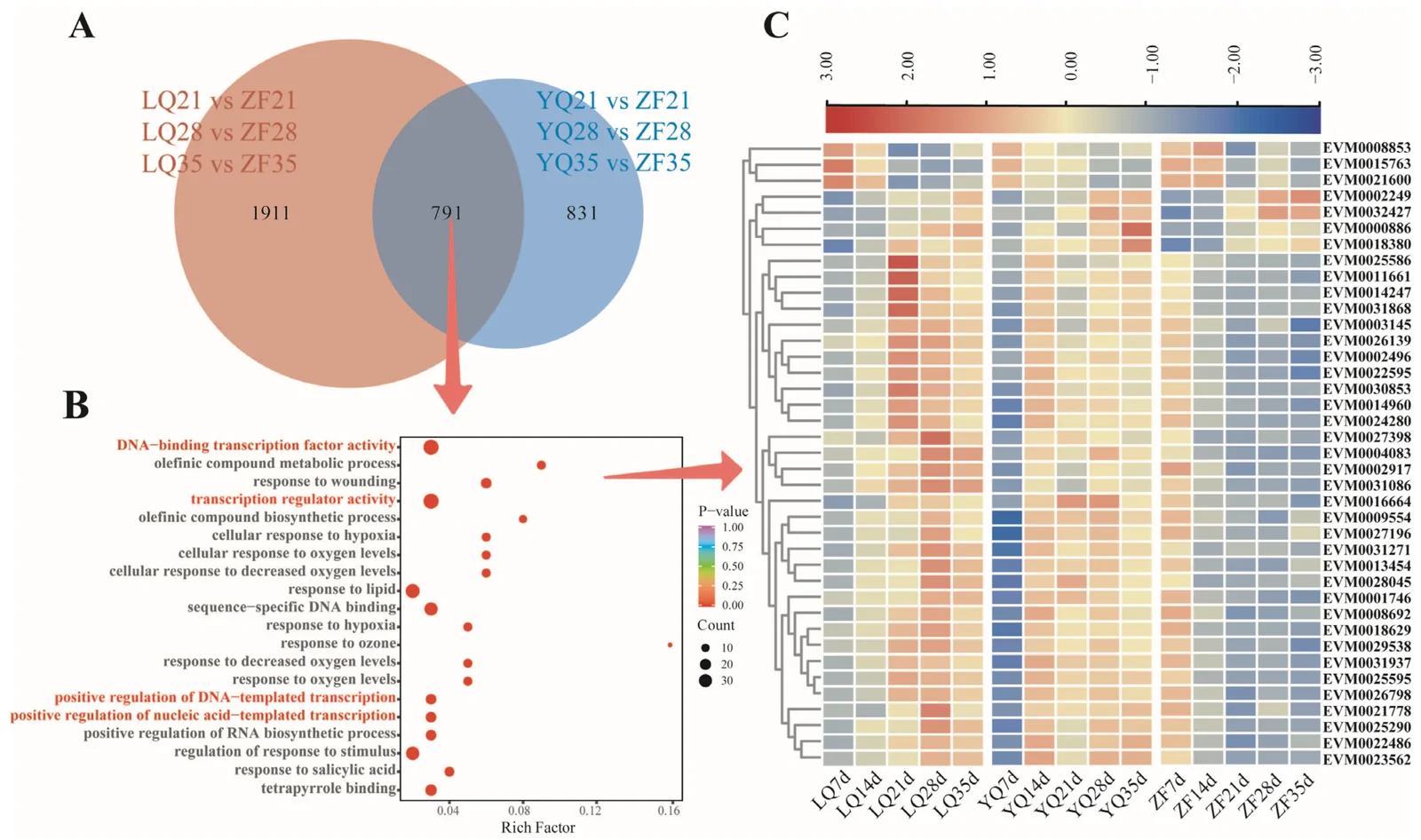
Shared DEGs and GO enrichment analysis. (**A**): Venn diagrams showing the DEGs identified in the LQ vs. ZF (red) and YQ vs. ZF (blue) comparisons at 21, 28, and 35 d after budbreak; 791 DEGs were shared between the two multi-stage comparison sets. (**B**): GO enrichment pathways for 791 DEGs; (**C**): Time-course expression levels of significantly enriched pathway-associated genes across the three experimental materials.

Morphological observations and previous cytological results suggested that 21 d after budbreak marked a critical stage of chestnut female flower development. To investigate the functions and interactions of associated DEGs, we combined genes identified in three pairwise cultivar comparisons at 21, 28, and 35 d. A total of 2702 DEGs were shared among the three LQ vs. ZF comparisons, whereas 1622 were shared among the corresponding YQ vs. ZF comparisons. Combining these sets identified 791 DEGs common to both cultivar contrasts (Figure 2A, Table S9). These genes were significantly enriched in two transcription factor activity pathways, one DNA-dependent transcription pathway, and one nucleic acid-dependent transcription pathway (Figure 2B). Genes associated with transcription factor activity also distinguished the two more-female cultivars from ZF (Figure 2C). Except for *EVM0008853*, *EVM0015763*, and *EVM0021600*, DEG expression generally increased in the more-female cultivars but peaked at 7 d and subsequently decreased in ZF. Thus, the more-female cultivars and ZF exhibited distinct transcriptional profiles during the middle and late stages of female flower development. These results are consistent with transcription factors acting as central links between gene expression and phenotype through activation or repression of downstream targets.

### 2.5. Screening of Transcription Factors

**Figure 3:**
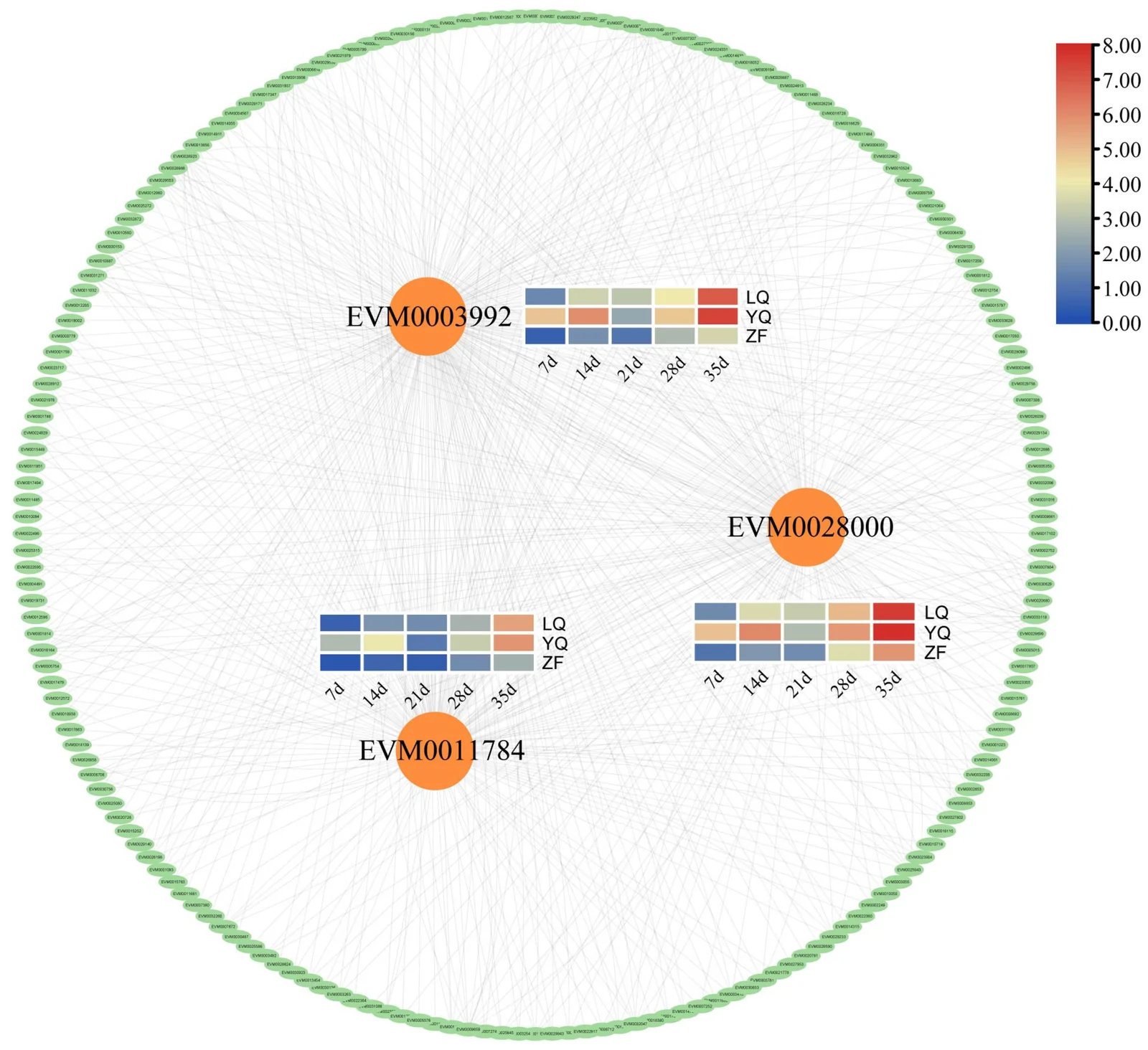
Predicted transcription factor regulatory network (gene-expression values are shown as log_2_-transformed FPKM). Orange circles denote the predicted transcription factors *EVM0003992*, *EVM0028000*, and *EVM0011784*. Adjacent color blocks show expression levels across samples, and the outer green circles denote genes with predicted binding sites for these transcription factors.

Mapping the 791 DEGs to Arabidopsis identified 20 genes encoding transcription factors: seven *AP2*, five *WRKY*, three *MADS-box*, two *NAC*, two *MYB*, and one *C2C2*. Several of these families, particularly *MADS-box* genes represented in the ABCDE model, have established roles in floral organ development. Motifs obtained from PlantTFDB were scanned against the promoter sequences of the 791 genes using the Binding Motif Scan module in TBtools-II v2.553 with default parameters. The analysis predicted that *MADS-box* transcription factors could bind the promoters of 191 DEGs. The three *MADS-box* genes identified in this analysis were expressed at higher levels in the more female cultivars than in ZF and generally increased during female flower development (Figure 3). These cultivar-specific patterns support the selection of *MADS-box* genes for further analysis of chestnut female flower development.

### 2.6. Analysis of the CmMADS Gene Family

#### 2.6.1. Identification, Physicochemical Properties, Phylogenetic Analysis, and Gene Structure of the CmMADS Family

We identified 60 *CmMADS* genes in chestnut and named them *CmMADS1*–*CmMADS60* according to their chromosomal positions. Their physicochemical properties are presented in Table S5. All encoded proteins were predicted to localize to the nucleus, consistent with their functions as transcription factors.

**Figure 4:**
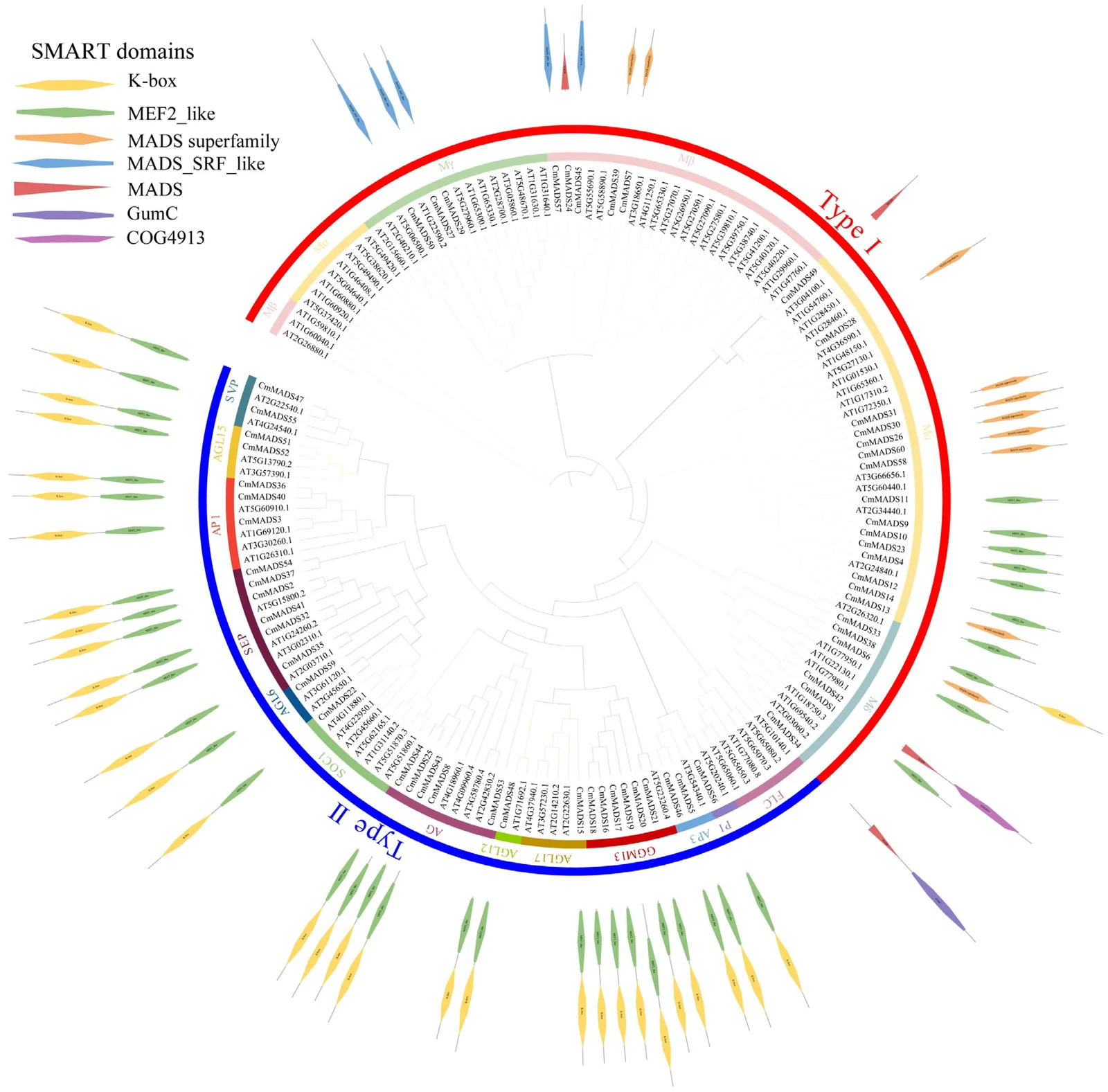
Phylogenetic analysis of the MADS-box gene family in *A. thaliana* and *C. mollissima*. The differently colored labels in the innermost circle indicate the MADS-box subfamilies. The outer red and blue circles represent Type I and Type II groups, respectively. Peripheral symbols denote SMART domains in CmMADS proteins; the corresponding domain names are shown in the upper-left corner.

Phylogenetic analysis classified the 60 CmMADS proteins into two major groups based on the SRF-TF domain (PF00319) and K-box domain (PF01486) (Figure 4). Twenty-nine proteins belonged to Type I (M-type), whereas 31 belonged to Type II (MIKC-type). Type I proteins were subdivided into the Mα, Mβ, Mγ, and Mδ subfamilies. Type II proteins comprised 13 subfamilies: FLC, PI, AP3, GGM13, AGL17, AGL12, AG, SOC1, AGL6, SEP, AP1, AGL15, and SVP. Conserved-domain distributions supported this classification. Conserved motifs and exon-intron structures also differed clearly between Type I and Type II genes (Figure S3). The greater structural complexity of Type II genes may be associated with their specialized functions in floral organ development.

#### 2.6.2. Chromosomal Localization and Collinearity Analysis of CmMADS Genes

**Figure 5:**
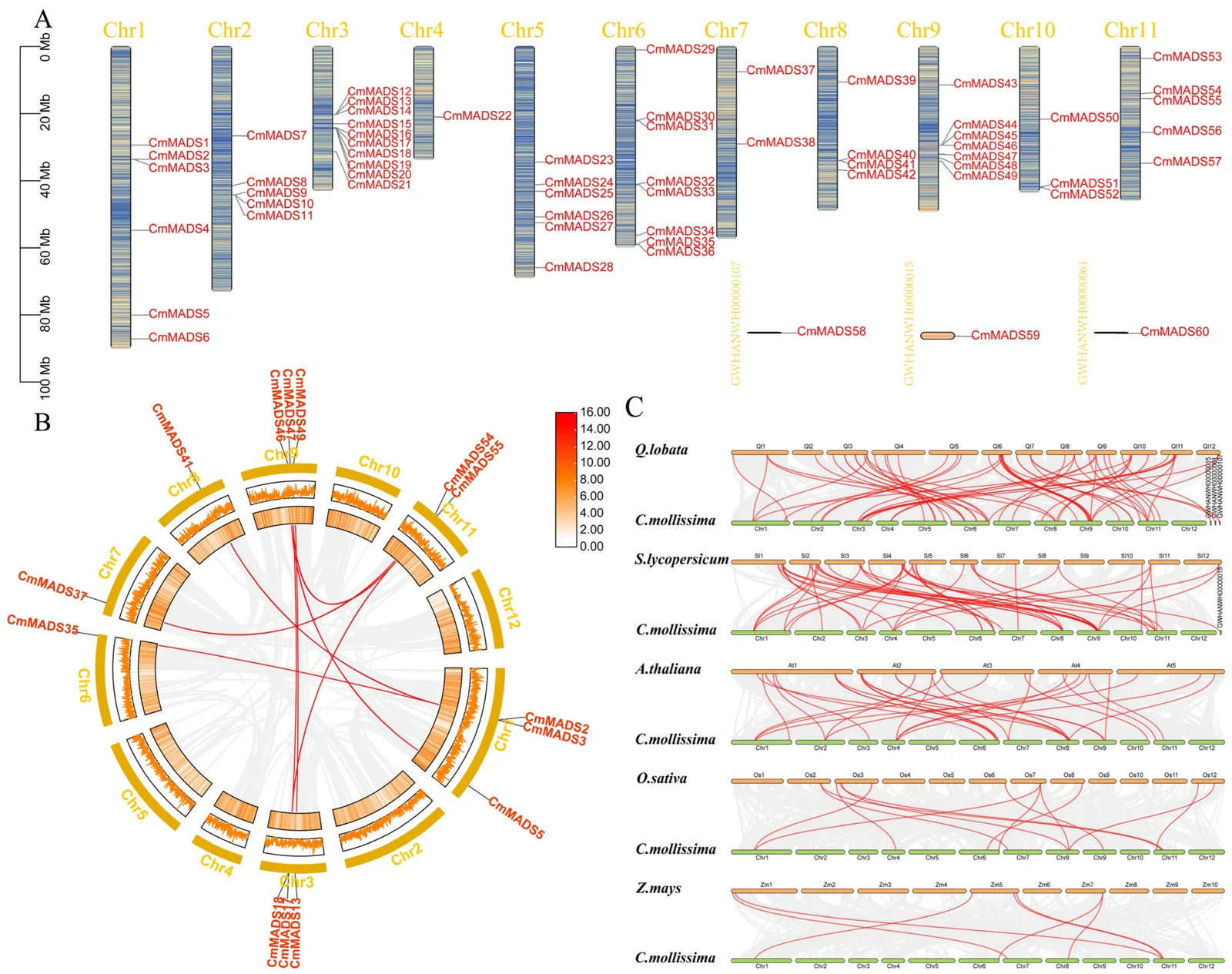
(**A**) Chromosomal distribution of *CmMADS* genes. Segment colors indicate gene density in the corresponding chromosomal regions. The three separate items represent genes located on scaffolds that were not assembled into larger fragments. (**B**) Intraspecific collinearity in *C. mollissima*. The middle red lines represent the 9 segmentally duplicated gene pairs. The inner circles represent chromosomal regions, with colors indicating regional gene density. (**C**) Synteny of *MADS-box* genes between *C. mollissima* and *Q. lobata*, *S. lycopersicum*, *A. thaliana*, *O. sativa*, and *Z. mays*. Gray lines indicate genomic collinear blocks; red lines indicate collinear gene pairs of *MADS-box*.

A chromosomal map was constructed from the genomic coordinates of the 60 *CmMADS* genes using TBtools. Fifty-seven genes were unevenly distributed across 11 chromosomes, whereas *CmMADS58*, *CmMADS59*, and *CmMADS60* were not assembled into large chromosomal scaffolds (Figure 5A). This uneven distribution reflects the diversity and complexity of the chestnut MADS-box family.

Intraspecific synteny analysis identified 11 duplicated gene pairs (Figure 5B). Nine segmentally duplicated pairs appeared to be the principal contributors to *CmMADS* family expansion, whereas two tandemly duplicated pairs may have contributed to functional diversification. All Ka/Ks ratios were less than 1 (Table S6), indicating that the duplicated genes underwent purifying selection. Duplication followed by purifying selection may therefore have promoted family expansion while maintaining functions related to floral development.

Interspecific collinearity analysis identified numerous conserved homologous genes between chestnut and oak (Figure 5C). Tomato and *Arabidopsis*, both dicots, showed the next highest levels of collinearity with chestnut. Homologous genes were particularly abundant on chromosomes 9, 6, and 3, respectively. Fewer and smaller collinear blocks were detected between chestnut and the monocots maize and rice.

#### 2.6.3. Cis-Acting Elements in CmMADS Promoters

**Figure 6:**
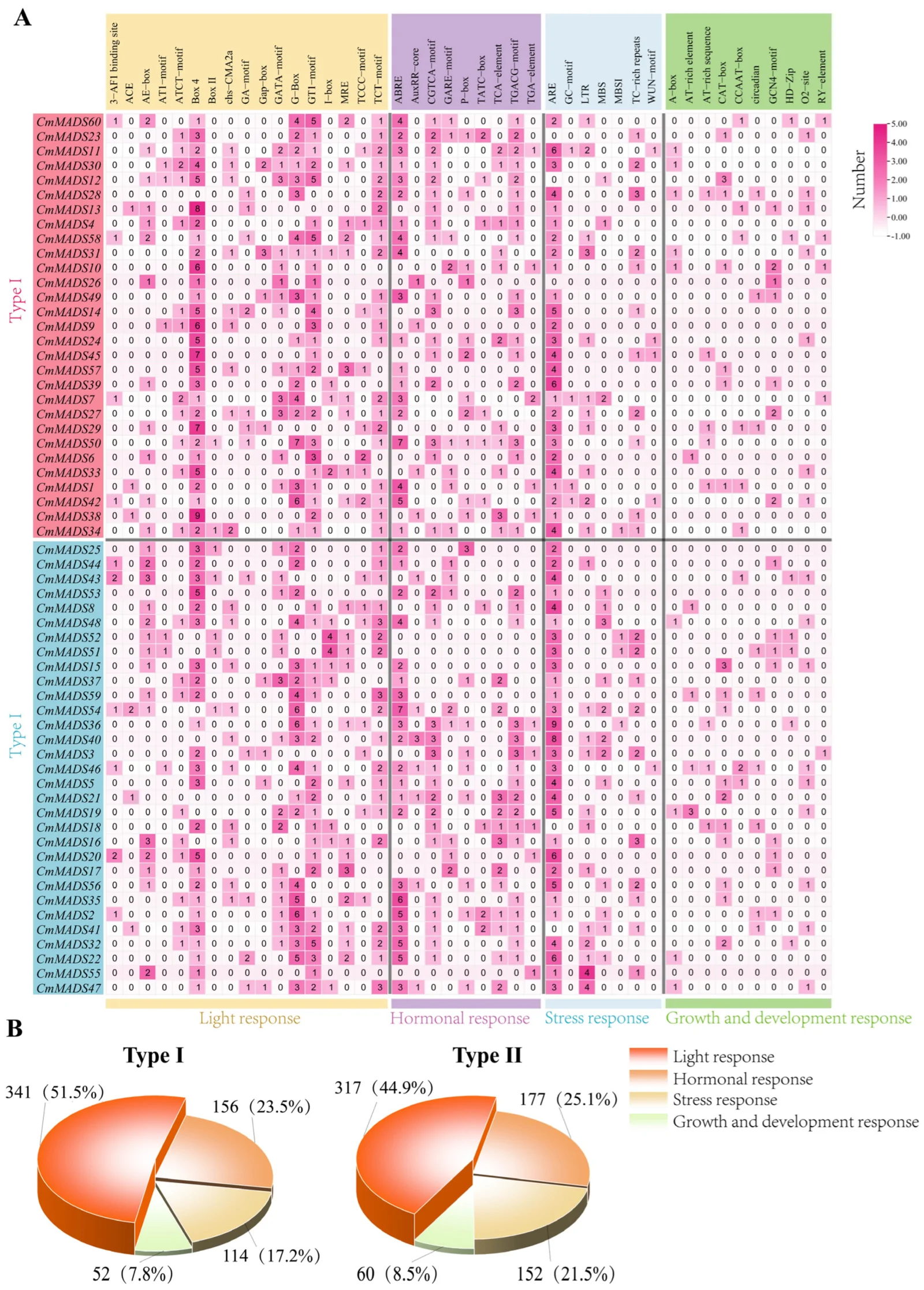
Cis-acting elements in *CmMADS* promoters. (**A**) Numbers and types of cis-acting elements. The text above the diagram indicates different types of cis-acting elements, while the text below shows the classification of all these response elements. (**B**) Numbers and percentages of response-element classes in Type I and Type II *CmMADS* genes.

Cis-acting element analysis showed that light-responsive elements were most abundant in Type I *CmMADS* promoters, accounting for more than 50% of the identified elements (Figure 6A). Their proportion was 6.5% lower in Type II promoters, accompanied by increases in hormone-, stress-, and growth-and-development-responsive elements. These distributions suggest that *CmMADS* genes may respond to both light and hormonal signals. Across the two major groups, light- and hormone-responsive elements accounted for approximately 50% and 25% of all elements, respectively, further supporting potential roles in light and hormone responses (Figure 6B).

We also identified several growth- and development-related cis-elements associated with endosperm and seed-specific functions. This pattern is consistent with established roles of *MADS-box* genes in reproductive development. The greater proportion of growth- and development-responsive elements in Type II promoters suggests that Type II *CmMADS* genes may have prominent roles in female flower differentiation and seed morphogenesis.

#### 2.6.4. Predicted CmMADS Protein Interaction Network

**Figure 7:**
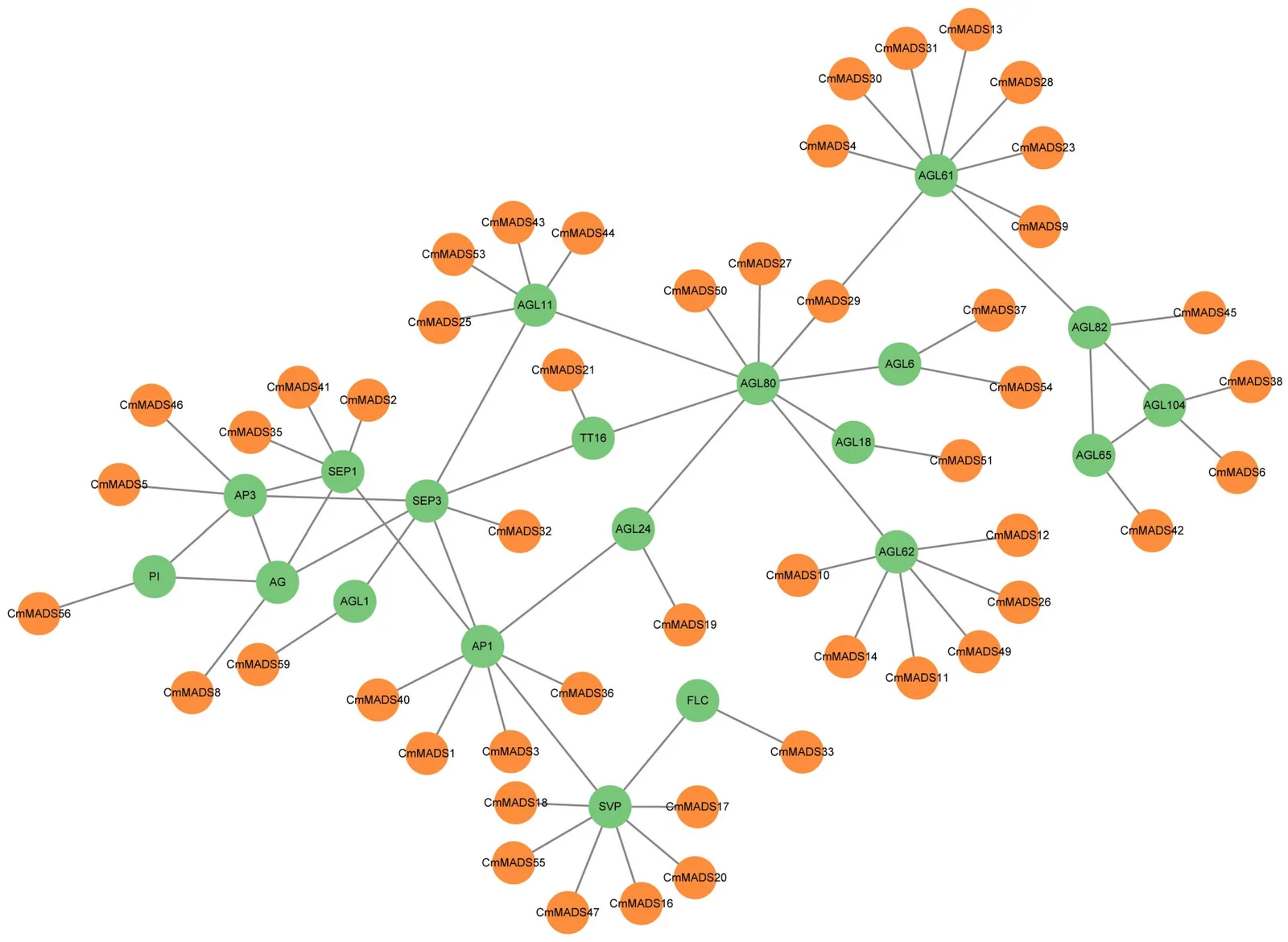
Protein interaction network predicted using *A. thaliana* homologs of CmMADS proteins. Both the green and orange circles represent the interaction network derived from *A. thaliana*; the orange CmMADS labels indicate the corresponding *C. mollissima* proteins identified by homology mapping.

Mapping the 60 CmMADS proteins to Arabidopsis identified 49 CmMADS proteins associated with 20 corresponding Arabidopsis proteins in the predicted interaction network (Figure 7). AP1, FLC, SVP, and AGL24 may regulate flowering; AGL62, AGL1, AGL11, AGL61, AGL80, and TT16 may contribute to ovule development; and SEP1, SEP3, AP3, PI, AG, AGL6, AGL65, AGL104, and AGL82 may function in flower development. The predicted network suggests that functionally related proteins may form complexes that jointly regulate floral organ development. The predicted network is based on interactions reported for *A. thaliana* homologs and has not been experimentally validated in chestnut tissues; it provides a reference for future functional studies of *C. mollissima* MADS-box proteins.

#### 2.6.5. Expression of CmMADS Genes in the Interaction Network

**Figure 8:**
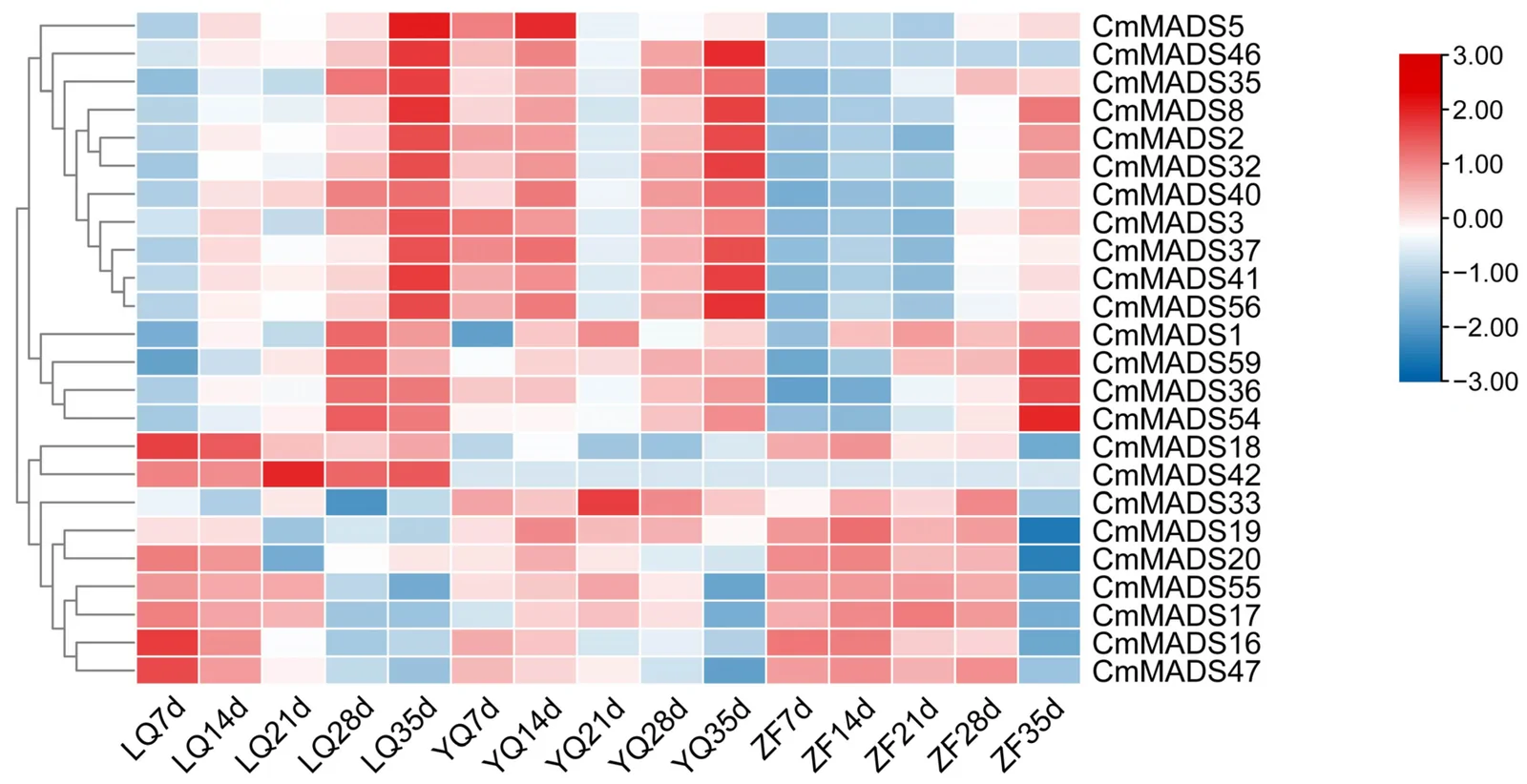
Differential expression of selected *CmMADS* genes in the predicted protein interaction network (Log_2_-transformed FPKM values represent gene expression levels).

We compared *CmMADS* expression across five stages of female flower development in LQ, YQ, and ZF using genes represented in the predicted interaction network (Figure 8; Table S10). Twenty-two *CmMADS* genes were expressed in all three cultivars, one was specific to LQ, and one was expressed in both LQ and YQ. The remaining 25 genes were not differentially expressed in the transcriptome.

Type II *CmMADS* genes generally showed higher expression during chestnut female flower development. Eleven genes (*CmMADS2*, *CmMADS3*, *CmMADS5*, *CmMADS8*, *CmMADS32*, *CmMADS35*, *CmMADS37*, *CmMADS40*, *CmMADS41*, *CmMADS46*, and *CmMADS56*) were highly expressed in LQ and YQ but expressed at lower levels in ZF. Four genes (*CmMADS1*, *CmMADS36*, *CmMADS54*, and *CmMADS59*) showed similar expression levels and trends across all three cultivars. Seven genes (*CmMADS16*, *CmMADS17*, *CmMADS19*, *CmMADS20*, *CmMADS33*, *CmMADS47*, and *CmMADS55*) were highly expressed in ZF but expressed at lower levels in LQ and YQ. These contrasting patterns identify genes potentially associated with the more-female phenotype in chestnut.

Genes expressed at high levels in the more female cultivars but at low levels in ZF generally increased during female flower development. Conversely, genes expressed at high levels in ZF but at low levels in the more female cultivars generally decreased.

#### 2.6.6. RT-qPCR Validation

Based on the expression profiles of the 24 genes and their predicted functional interactions, we selected *CmMADS* genes associated with four representative proteins (AG, AP1, PI, and SVP) for RT-qPCR validation. *CmMADS8*, *CmMADS36*, and *CmMADS56* generally increased during female flower development, suggesting that they may be positively associated with this process. *CmMADS16* decreased and was expressed at a higher level in ZF, suggesting a possible negative association with female flower development.

**Figure 9:**
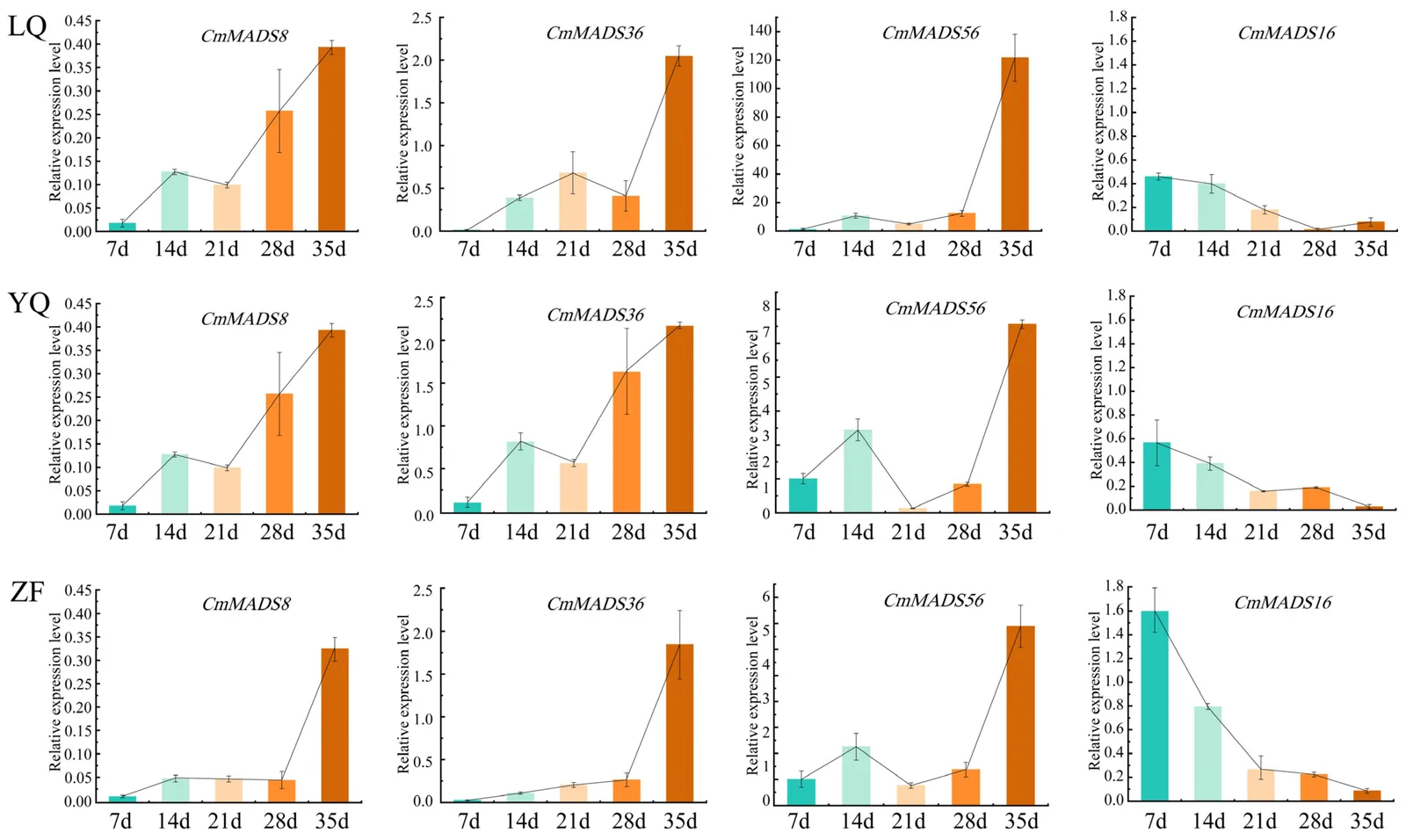
Expression levels of four *CmMADS* genes during female flower development in LQ, YQ, and ZF. The *y*-axis shows the gene expression levels measured by RT-qPCR, and the *x*-axis shows developmental stages (days after budbreak). The error bars represent the mean ± standard deviation (SD).

According to the predicted functional interactions among gene subfamilies, *CmMADS* members from different subfamilies exhibit potential associations that may correlate with the formation of floral organ differentiation phenotypes. Expression trends measured by RT-qPCR were consistent with the RNA-seq data. These findings indicate that *MADS-box* genes are associated with early female flower development, and the expression changes in this gene family may correlate with the earlier and more complete differentiation characteristics of female flowers in LQ and YQ (Figure 9).

## 3. Discussion

### 3.1. Phenotypic Observations and Transcriptomic Analysis of Female Flower Formation in C. mollissima

Sex differentiation in dioecious plants generally follows two developmental routes. In some species, early flowers tend toward hermaphroditic differentiation, after which pistil or stamen primordia degenerate to produce unisexual flowers [45]. In others, stamens and pistils differentiate independently without subsequent organ degeneration or abortion [46]. Chestnut produces both male catkins and mixed inflorescences. Mixed inflorescences contain female flower clusters and elongated male inflorescences, and the female flowers are enclosed by spiny bracts, typically in clusters of three. Female flower formation strongly influences chestnut reproductive success and yield. Previous transcriptomic studies of mixed chestnut inflorescences have focused mainly on individual cultivars [8,47]. Here, we systematically compared the two more female cultivars LQ and YQ with the control ZF.

Morphological and transcriptomic evidence jointly identified 21 d after budbreak as a key point at which cultivar differences in sex differentiation emerged. LQ and YQ continued to differentiate normally and largely completed early female flower development by 28 d, whereas ZF showed delayed or impaired development. Consistent with this phenotype, the LQ vs. ZF comparison showed a sharp increase to 2223 DEGs at 21 d. Previous cytological observations further indicated that hermaphroditic inflorescences became visible at 28 d. Central floral primordia were more highly differentiated in the LQ and YQ than in ZF, potentially favoring female flower-cluster formation [2]. CTK and GA concentrations in LQ and YQ also peaked at 28 d, coinciding with stamen and pistil primordium differentiation. Together, these observations support the biological importance of this developmental stage.

Analysis of DEGs at 21, 28, and 35 identified 791 genes consistently differentially expressed between the more female cultivars and ZF. These genes were enriched in transcription factor activity, DNA-dependent transcription, and nucleic acid-dependent transcription, supporting a central role for transcriptional regulation in female flower differentiation. Twenty of the genes encoded predicted transcription factors, 15% of which belonged to the MADS-box family. Their high expression in the LQ and YQ floral organ suggests that *MADS-box* genes may contribute to female flower differentiation and development. This interpretation is consistent with reports of higher *MADS-box* expression in chestnut floral organs than in leaves and cotyledons [6,48].

### 3.2. Identification of the CmMADS Transcription Factor Family

Transcription factors are central regulators of plant growth and development, particularly floral development [49]. MADS-box, AP2, WRKY, MYB, and bHLH are among the most extensively studied transcription factor families.

*MADS-box* transcription factors regulate environmental responses, cell differentiation, organ identity, and floral organ formation. Most functional genes in the classical ABCDE model, including AG, belong to the MADS-box family and regulate floral organ identity and sex differentiation [50]. Characterizing *CmMADS* genes may therefore clarify mechanisms governing female flower development and sex differentiation in chestnut floral buds. In this study, transcriptome and GO enrichment analyses identified *CmMADS* transcription factors as candidates. This finding is consistent with the results of Wang [47] and supports a close association between the CmMADS family and chestnut female flower development.

We systematically identified 60 *CmMADS* genes. Their encoded proteins showed diverse physicochemical properties and were predicted to localize to the nucleus, consistent with their transcription factor functions. The genes were classified into Type I (M-type) and Type II (MIKC-type) groups. The Mδ subfamily was assigned to Type I because it lacks a conserved K-box domain. The number of MIKC-type members was 13.5% greater in chestnut than in Arabidopsis, similar to the pattern reported in *Camellia chekiangoleosa* [51]. This difference may reflect a lower duplication rate among Type I genes and a higher duplication rate among Type II genes.

### 3.3. Evolution of the CmMADS Gene Family

Gene duplication, including whole-genome, segmental, and tandem duplication, provides genetic material for evolutionary innovation [34,35,36,47]. We detected 11 duplicated *CmMADS* pairs, nine of which were segmental. Segmental duplication may therefore have contributed substantially to family expansion, consistent with reports in *Salvia miltiorrhiza*, *Rhododendron hainanense*, and *Sechium edule* [51,52,53].

Whole-genome duplication, an important driver of gene-family expansion [38], was not identified in the CmMADS family, which may explain its relatively small size. All Ka/Ks ratios calculated for duplicated pairs were less than 1, indicating purifying selection [34]. Purifying selection generally promotes functional conservation, whereas gene duplication can facilitate functional diversification. Together, these processes may have supported complex regulation of chestnut floral development while maintaining a conserved core of MADS-box functions.

Cross-species synteny was strongest between chestnut and oak, both members of Fagaceae, followed by the dicots tomato and *Arabidopsis* [39]. Reduced synteny with rice and maize suggests that many conserved pairs arose or were retained after the monocot–dicot divergence. The strong homology between chestnut and Arabidopsis supports the use of Arabidopsis as a comparative model, although chestnut-specific functions require direct validation.

### 3.4. Gene Structure and Potential Regulatory Functions

Type II *CmMADS* genes contained more and longer introns than Type I genes. Greater intron number and length can increase opportunities for alternative splicing and influence gene expression or protein activity [54,55,56]. Multiple introns provide additional splice-site combinations that may increase transcript isoform diversity and regulatory complexity in plants. Most Mα, Mβ, and Mγ genes contained no introns or a single intron, whereas Type II genes typically contained four to eight. It has been suggested that genes with super large introns and transposons are more highly expressed [57,58]. In addition, MIKC-type genes regulate floral organ development, flowering duration, and sex determination [59]. Thus, the greater structural complexity of Type II *CmMADS* genes, reflected by richer intron composition, may support functional diversification underlying floral-related developmental processes, consistent with observations in *C. chekiangoleosa*, where intron-rich Type II MIKC^C^ genes showed higher expression and prominent roles in reproductive-organ regulation [52].

Light-responsive elements accounted for the largest proportion of cis-regulatory elements in all *CmMADS* promoters, but their distribution differed between Type I and Type II genes. Type II promoters contained a lower proportion of light-responsive elements and more hormone-, stress-, and growth-and-development-responsive elements than Type I promoters. Floral organ development is jointly regulated by genetic factors, environmental signals such as photoperiod and temperature, and plant hormones [44]. Together with the greater exon-intron complexity of Type II genes, these patterns support a regulatory model. *CmMADS* genes may sense environmental and endogenous hormonal signals through promoter cis-elements and respond transcriptionally. The resulting MADS-box proteins may form homo- or heterodimers or multiprotein complexes that regulate downstream targets involved in female flower and reproductive organ development. This model remains to be tested experimentally.

Hormone signaling may be particularly relevant to female flower differentiation. Earlier work from our group associated high CTK and GA concentrations during pistil primordium differentiation with the more-female phenotype [2]. Other chestnut studies have implicated hormones in floral differentiation through transcriptional regulation [5,6,7]. The combined evidence favors a regulatory network integrating transcription factors with environmental and hormonal inputs rather than a single pathway. Hormone signaling is also amenable to horticultural manipulation.

### 3.5. Expression-Pattern Analysis and Functional Prediction of CmMADS Genes

Integrating predicted protein interactions with transcriptome data identified 24 differentially expressed *CmMADS* genes. Eleven genes highly expressed in LQ and YQ were associated with SEP1, SEP3, PI, AP3, AG, and AGL6, which regulate flower development and floral organs in Arabidopsis. Seven genes highly expressed in ZF but expressed at lower levels in LQ and YQ were associated with AGL24, SVP, and FLC. These proteins act as transcriptional repressors in the autonomous flowering pathway, and SVP delays Arabidopsis flowering by repressing flowering-pathway integrator genes. Four genes showed similar expression patterns across the three cultivars and were associated with AP1, AGL1, and AGL6, which participate in endosperm development and floral-meristem regulation. These contrasting expression patterns suggest coordinated regulation of female flower development and possible contributions to the LQ and YQ phenotype. RT-qPCR validation of four representative genes confirmed the major transcriptome expression trends. The identified *CmMADS* genes provide candidates for functional studies of female flower and floral organ development in chestnut.

### 3.6. Future Prospects

This study assessed transcript-level expression and predicted gene functions. Future studies should expand the germplasm panel and test candidate *CmMADS* functions through overexpression or silencing, promoter analysis, and protein-interaction assays. Notably, the transient-transformation systems for chestnut still face technical bottlenecks, so heterologous-system assays may serve as an intermediate step for subsequent functional verification.

## 4. Materials and Methods

### 4.1. Plant Material Sampling and Phenotypic Observation

The experimental site was located in the chestnut germplasm orchard of Hebei Normal University of Science and Technology in Changli, Qinhuangdao, Hebei Province (119.52° E, 39.93° N). The region has a warm-temperate, semi-humid monsoon climate, with a mean annual temperature of 11.7 °C and annual precipitation of 600–800 mm. Grafted ten-year-old trees of the more-female Yanshan chestnut cultivars ‘Yanqiu’ (YQ) and ‘Longquanzhuang No. 2’ (LQ) and the control ‘Yanshanzaofeng’ (ZF) were managed uniformly. Three healthy trees per cultivar served as biological replicates. At 7, 14, 21, 28, and 35 d after budbreak, ten moderately growing shoots bearing mixed inflorescences were collected from the outer canopy of each tree. The three trees provided separate biological samples for each cultivar-stage, yielding 45 biological sample. Samples containing mixed inflorescences or female flower clusters were immediately frozen in liquid nitrogen, transported to the laboratory, and stored at −80 °C for transcriptome sequencing, gene cloning, and RT-qPCR analysis.

Morphological observations were conducted from mid-to-late April, beginning at floral-bud break. Five shoots were selected from each of the three trees per cultivar, yielding a total of 15 shoots that were tagged for ongoing morphological monitoring. Flower buds, mixed inflorescences, and female flowers on fruiting shoots were photographed, and their developmental status was recorded. Representative images were selected for presentation.

### 4.2. Transcriptome Analysis

Total RNA was extracted from all samples and assessed by agarose gel electrophoresis and a Qsep400 Bioanalyzer (RIN ≥ 7). mRNA libraries were prepared using the NEBNext Ultra RNA Library Prep Kit (NEB, Ipswich, MA, USA) and sequenced by Metware Biotechnology Co., Ltd. (Wuhan, China) on an Illumina NovaSeq 6000 platform with 150 bp paired-end reads. The raw reads are available in the NCBI Sequence Read Archive under BioProject accession PRJNA1522526 and SRA Study accession SRP733067. The BioSample accessions are SAMN62895590-SAMN62895634, and the SRA Run accessions are SRR40488672-SRR40488716 (The accession numbers corresponding to the 45 samples are provided in Table S12). The reference genome and gene-model annotation were downloaded from the National Genomics Data Center (https://ngdc.cncb.ac.cn/gwh, accessed on 15 November 2025). Adapters and low-quality reads were removed to obtain clean reads; Q20 and Q30 statistics are reported in Table S2. Clean reads were mapped to the *C. mollissima* reference genome using HISAT2. Transcripts were assembled and expression levels estimated using StringTie v2.1.6 [60]. Gene expression was quantified as fragments per kilobase of transcript per million mapped reads (FPKM) using featureCounts v2.0.3. FPKM values were used solely for expression visualization and descriptive comparisons. Differential expression analysis was performed using the DESeq2 package (v1.22.1) in R (v4.2.2), with raw count matrices generated by featureCounts as the input data, applying a threshold of |log2 fold change| ≥1 and false discovery rate (FDR) <0.05. Gene Ontology (GO) enrichment analysis was performed using clusterProfiler v4.6.0. Enrichment significance was calculated using the hypergeometric test. To control for multiple testing, the Benjamini–Hochberg procedure was applied to calculate False Discovery Rate (FDR) adjusted *p*-values. GO terms with FDR <0.01 were considered statistically significantly enriched. The genes were annotated using the SwissProt, GO, and Pfam databases, and the results are presented in Table S8 as the background gene set for enrichment analysis. Enrichment plots were generated using Metware Cloud (https://cloud.metware.cn, accessed on 20 November 2025). Principal component analysis (PCA) was performed using Genesis v1.8.1 [61].

### 4.3. Transcription Factor Prediction and Candidate Gene Screening

DEGs annotated as transcription factors in SwissProt were considered putative transcriptional regulators. Their amino acid sequences were extracted from the protein FASTA file and submitted to PlantTFDB (https://planttfdb.gao-lab.org/, accessed on 3 December 2025) to predict transcription factor families and retrieve binding motifs. The binding motif file of TFs and promoter sequences of candidate genes were further imported into the Fimo: Binding Motif Scan module of TBtools-II to predict potential transcription-factor-binding sites in the 2000 bp upstream regions(*p* ≤ 1 × 10^−4^) [62]. Cytoscape v3.9.1 was used for network visualization. Genes with predicted binding sites for MADS-box transcription factors and the corresponding IDs of binding motifs are listed in Table S11.

### 4.4. Identification of CmMADS Family Members, Gene Structure Analysis, and Phylogenetic Analysis

MADS-box protein sequences from *C. mollissima* and *A. thaliana* were obtained from the National Genomics Data Center (https://ngdc.cncb.ac.cn/gwh, accessed on 7 December 2025) and The Arabidopsis Information Resource (TAIR; https://www.arabidopsis.org/BROWSE/genefamly/MADSlike.jsp, accessed on 7 December 2025).

Two complementary strategies were used to identify chestnut MADS-box genes: local BLAST searches in TBtools II [63] and Hidden Markov Model (HMM) searches in HMMER v3.0 (http://hmmer.org/, accessed on 8 December 2025). Reference MADS-box proteins from *C. mollissima* and *A. thaliana* were first used in local BLAST searches against the chestnut transcriptomic database with an E-value threshold of 1 × 10^−5^. HMM profiles for the MADS domain (PF00319) and K-box domain (PF01486) were then downloaded from Pfam and searched against the same database using HMMER with an E-value threshold of 1 × 10^−5^. Results from both approaches were combined, and redundant sequences were removed. Conserved domains were verified using SMART and NCBI CDD, and conserved motifs were identified using MEME. Candidates with incomplete MADS domains or protein lengths below 100 amino acids were excluded as possible pseudogenes or incomplete sequences. Type I and Type II genes were classified according to the absence or presence, respectively, of a conserved C-terminal K-box domain [13,17,18]. Full-length *C. mollissima* and *A. thaliana* MADS-box protein sequences were aligned in ClustalW with default parameters. A neighbor-joining tree was constructed in MEGA11 with 1000 bootstrap replicates and visualized using iTOL (https://itol.embl.de/, accessed on 14 December 2025).

### 4.5. Physicochemical Properties, Chromosomal Localization, and Collinearity Analysis

The physicochemical properties of CmMADS proteins, including amino acid number, molecular weight, and isoelectric point, were calculated using Protein Parameter Calc in TBtools. Subcellular localization was predicted using PLANT-mPLoc. Gene coordinates were obtained from the chestnut genome database, and duplication events were analyzed using the built-in One Step MCScanX module of TBtools software. Genomic data for *C. mollissima*, *A. thaliana*, *Q. lobata*, *S. lycopersicum*, *O. sativa*, and *Z. mays* were used for interspecific collinearity analysis. Synonymous (Ks) and nonsynonymous (Ka) substitution rates and Ka/Ks ratios were calculated using the Simple KaKs_Calculator module of TBtools software [64] using four CPU cores; all other parameters were set to default values.

### 4.6. Cis-Acting Element Analysis and Protein Interaction Network Prediction

The 2000 bp regions upstream of the CmMADS genes were extracted as promoter sequences. Cis-acting elements were predicted using PlantCARE and visualized in TBtools. Because STRING does not contain a *C. mollissima* database, CmMADS proteins were mapped to their *A. thaliana* homologs and used to construct a predicted interaction network in STRING. The network was visualized using Cytoscape v3.9.1.

### 4.7. Gene Expression Analysis and RT-qPCR Validation

cDNA was synthesized from 1000 ng of total RNA in a 20 µL reaction using the PC18-TRUEscript 1st Strand cDNA Synthesis Kit (Aidlab, Beijing, China) according to the manufacturer’s instructions. RT-qPCR was performed using a 2× SYBR Green I kit (Aidlab, Beijing, China) on a Bio-Rad CFX96 Real-Time PCR System in a 20 µL reaction. The cycling conditions were 98 °C for 3 min; 38 cycles of 98 °C for 30 s, 58 °C for 30 s, and 72 °C for 90 s; and a final extension at 72 °C for 5 min. Three biological replicates and three technical replicates were analyzed. *C. mollissima* Actin (*CmActin*) served as the reference gene [65], and relative expression was calculated using the 2^−ΔΔCt^ method. Primers were designed using Primer Premier v5.0 and are listed in Table S7. The RT-qPCR primer efficiency was determined using the standard-curve method [66,67]. The reference gene was amplified across all tested samples (Figure S4). In this study, we did not employ algorithmic approaches to systematically evaluate the expression stability of the reference gene *CmActin* under different treatment conditions, nor did we archive the negative control results. These methodological shortcomings might, to some extent, affect the accuracy of the quantitative results. However, NTCs were included in every run and showed no amplification, and the results were reproducible; therefore, these limitations are unlikely to alter the main conclusions of this study.

### 4.8. Statistical Analysis

Statistical analyses were performed in Origin 2024, and final figures were prepared in Adobe Illustrator 2022. Data are presented as the mean ± standard deviation (SD).

## 5. Conclusions

Female flower development in chestnut is a dynamic process, with the greatest cultivar divergence emerging at approximately 21 d after budbreak. The more-female cultivars had largely completed early morphogenesis by 28 d. Transcriptome analysis identified *MADS-box* transcription factors as candidates associated with this developmental transition. We identified 60 chestnut *MADS-box* genes, comprising 29 Type I and 31 Type II genes. Type II genes contained more and longer introns than Type I genes, which may contribute to their higher expression. Integrating predicted protein interactions with transcriptome expression patterns identified 11 *CmMADS* genes that were highly expressed and generally increased in the more-female cultivars. These genes belonged to the SEP, PI, AP3, AG, and AGL6 subfamilies. Seven genes from the GGM13, SVP, and Mδ subfamilies were highly expressed in ZF and decreased during development. These contrasting patterns identify candidate regulators associated with differences in female flower differentiation between more-female and control cultivars. Functional experiments are required to establish their specific roles.

## Data Availability

The data presented in this study were submitted to the NCBI Sequence Read Archive (accession number: PRJNA1522526).

## Supplementary Materials

The following supporting information can be downloaded at: https://www.mdpi.com/article/10.3390/ijms27208994/s1.
