# Supplementary material for: Genome-Wide Identification of the Castanea mollissima MADS-Box Gene Family and Its Potential Roles in Female Flower Development Based on Transcriptomic and Morphological Analyses
Source: Int J Mol Sci. 2026 Oct 10;27(20):8994. doi: 10.3390/ijms27208994 (PMC13705099; doi:10.3390/ijms27208994)
Supplement: Supplementary file 1 [file ijms-27-08994-s001.zip › Supplementary Files.pdf]

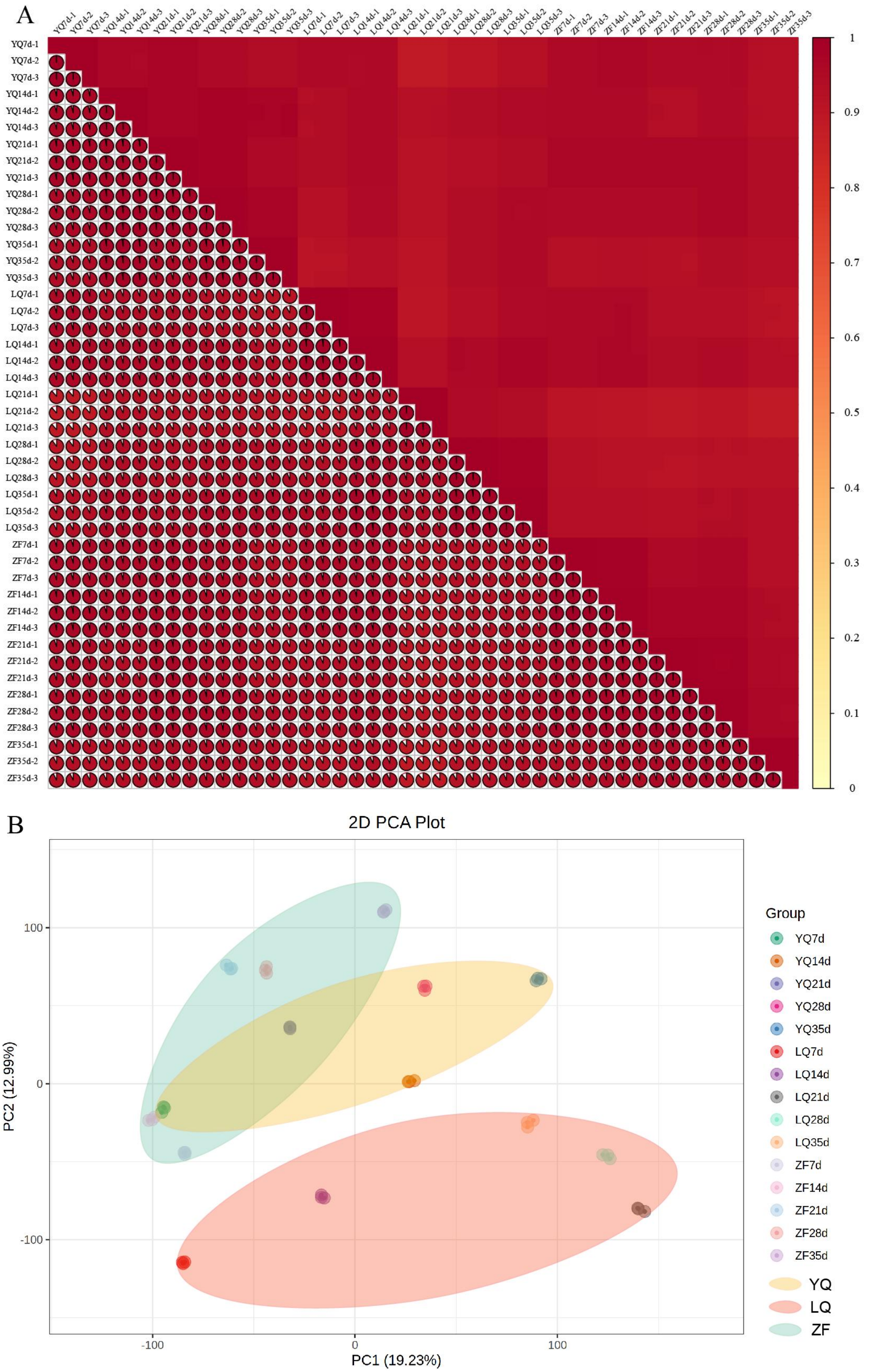


**Figure S1.** Sample correlation analysis；(A)Sample correlation diagram；(B)Sample PCA plot

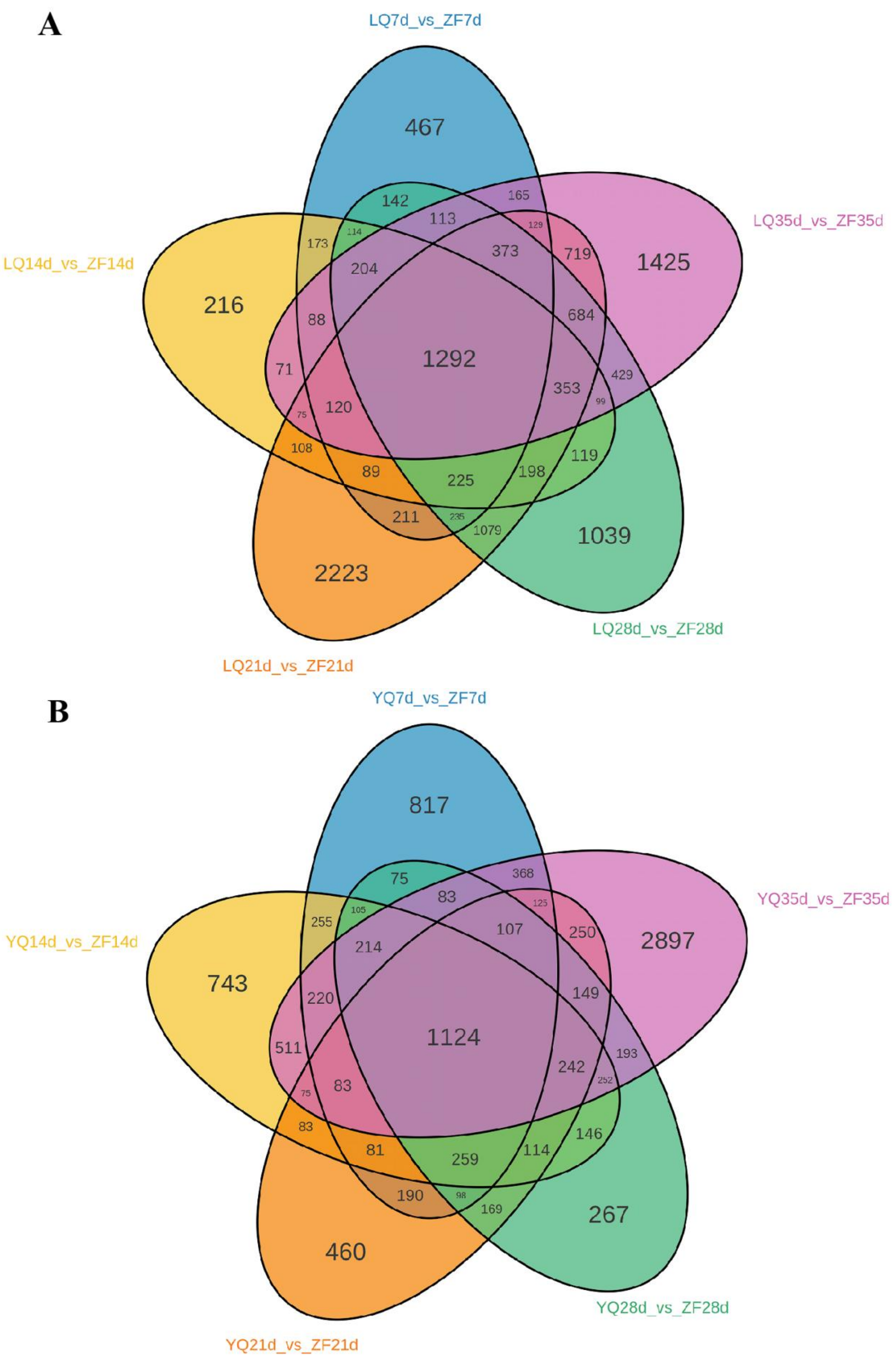


**Figure S2.** The Veen diagrams of differentially expressed genes；(A)Differential gene between LQ and ZF during the same period；(B)Differential gene between YQ and ZF during the same period

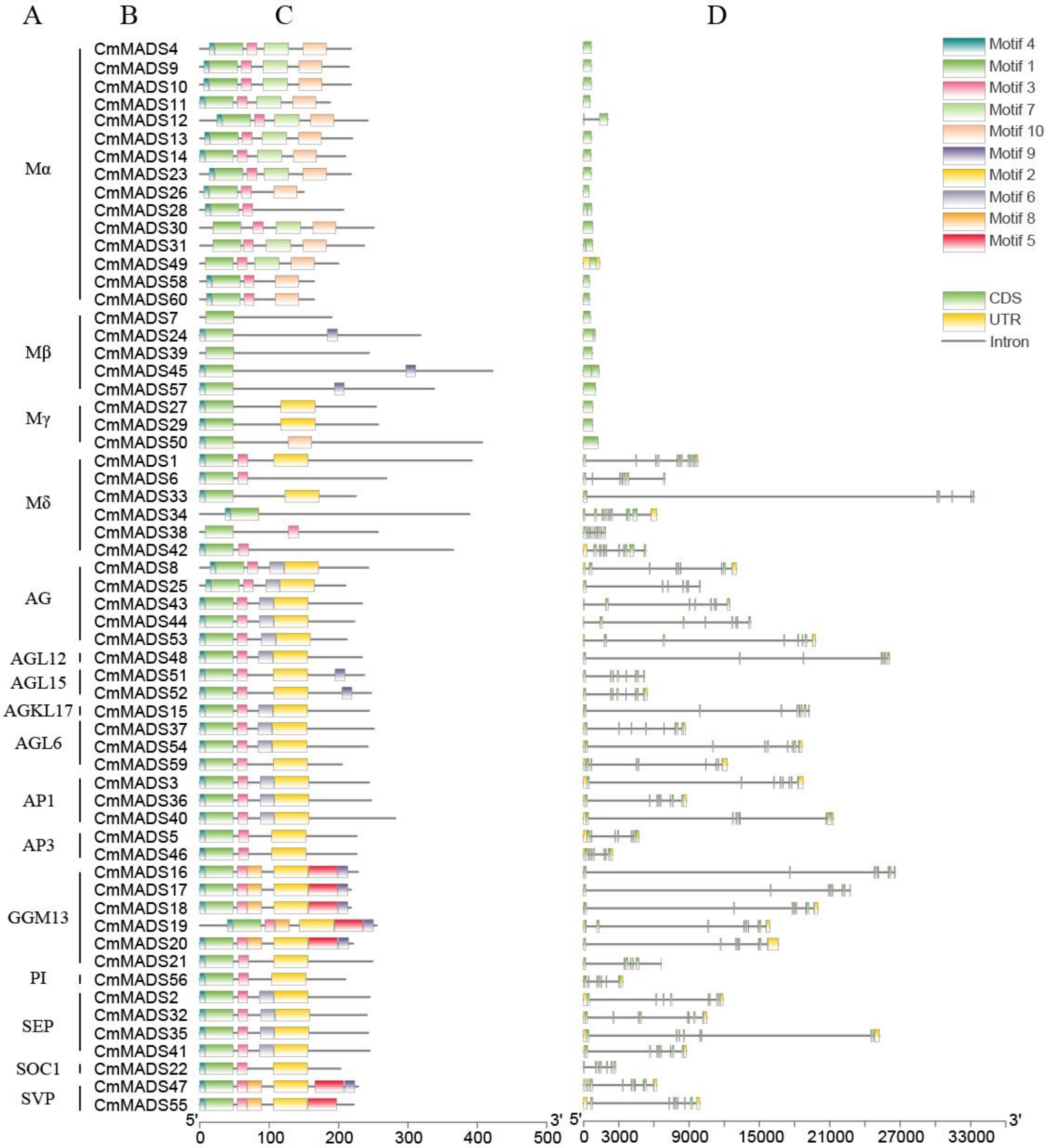


**Figure S3.** Gene structure and conserved motif of *CmMADS* family genes (A: Subfamily name; B：Gene name; C: Conserved motif of *CmMADS* family genes; D: Gene structure of *CmMADS* family genes)

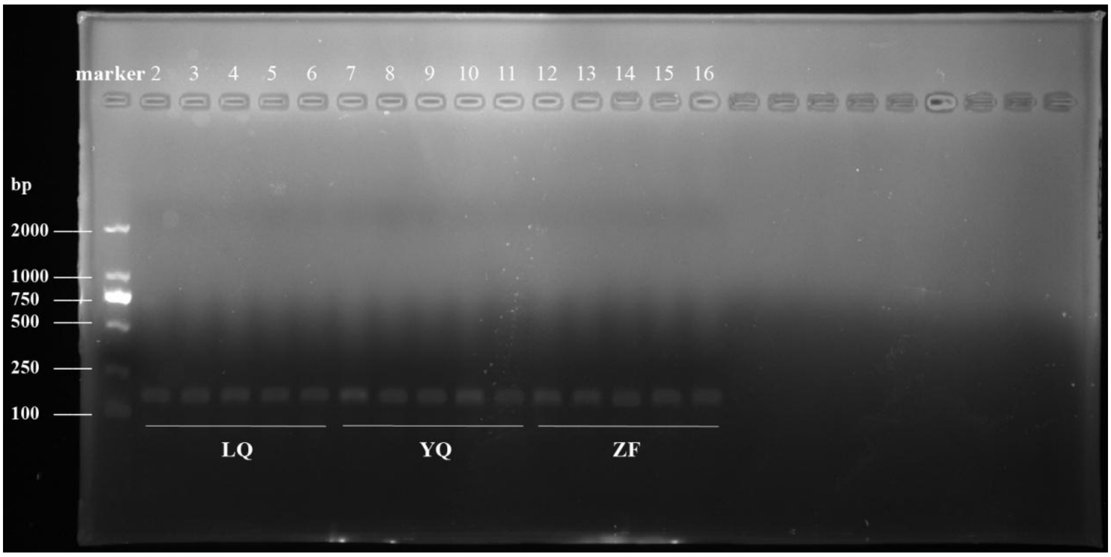


**Figure S4.** Agarose gel electrophoresis of PCR products for the internal reference genes in the test materials. **Note:**Lanes 2–6 are designated as LQ7-35d, lanes 7–11 as YQ7-35d, and lanes 12–16 as ZF7-35d.

**Table S1.** Comparison table of buds,inflorescences and female flower clusters phenological period for YQ, LQ and ZF

| | Date of bud burst | Sampling time | | | | |
|---|---|---|---|---|---|---|
| | | 7d | 14d | 21d | 28d | 35d |
| LQ | 4.19 | 4.26 | 5.3 | 5.10 | 5.17 | 5.24 |
| YQ | 4.18 | 4.25 | 5.2 | 5.9 | 5.16 | 5.23 |
| ZF | 4.15 | 4.22 | 4.29 | 5.6 | 5.13 | 5.20 |

**Table S2.** Statistics of Clean reads

| Sample | Raw Reads | Clean Reads | Clean Base(G) | Error Rate(%) | Q20(%) | Q30(%) | GC Content(%) |
|---|---|---|---|---|---|---|---|
| YQ7d-1 | 68306232 | 67147238 | 10.07 | 0.01 | 98.72 | 96 | 44.03 |
| YQ7d-2 | 67009128 | 65600492 | 9.84 | 0.01 | 98.82 | 96.28 | 44.37 |
| YQ7d-3 | 78770388 | 77273600 | 11.59 | 0.01 | 98.77 | 96.16 | 43.91 |
| YQ14d-1 | 49269028 | 48382002 | 7.26 | 0.01 | 98.65 | 95.78 | 43.72 |
| YQ14d-2 | 74063572 | 72746830 | 10.91 | 0.01 | 98.64 | 95.75 | 43.71 |
| YQ14d-3 | 71138660 | 69885270 | 10.48 | 0.01 | 98.71 | 95.98 | 43.62 |
| YQ21d-1 | 64749872 | 63780888 | 9.57 | 0.01 | 98.69 | 95.94 | 43.97 |
| YQ21d-2 | 65226164 | 64242790 | 9.64 | 0.01 | 98.59 | 95.61 | 44.07 |
| YQ21d-3 | 70288000 | 69249104 | 10.39 | 0.01 | 98.69 | 95.93 | 44.02 |
| YQ28d-1 | 58886104 | 58218162 | 8.73 | 0.01 | 98.64 | 95.7 | 43.78 |
| YQ28d-2 | 56642550 | 55829626 | 8.37 | 0.01 | 98.62 | 95.71 | 43.84 |
| YQ28d-3 | 73540142 | 72519446 | 10.88 | 0.01 | 98.68 | 95.87 | 43.74 |
| YQ35d-1 | 71076244 | 69978808 | 10.5 | 0.01 | 98.63 | 95.74 | 43.58 |
| YQ35d-2 | 59358444 | 58479320 | 8.77 | 0.01 | 98.76 | 96.08 | 43.35 |
| YQ35d-3 | 79896106 | 78730958 | 11.81 | 0.01 | 98.78 | 96.2 | 43.64 |
| LQ7d-1 | 53653356 | 52598542 | 7.89 | 0.01 | 98.63 | 95.72 | 44.13 |

| | | | | | | | |
|---|---|---|---|---|---|---|---|
| LQ7d-2 | 75918096 | 74403658 | 11.16 | 0.01 | 98.69 | 95.89 | 44 |
| LQ7d-3 | 76834994 | 75190950 | 11.28 | 0.01 | 98.76 | 96.12 | 44.2 |
| LQ14d-1 | 66994928 | 66079704 | 9.91 | 0.01 | 98.68 | 95.88 | 44.08 |
| LQ14d-2 | 65687326 | 64886704 | 9.73 | 0.01 | 98.58 | 95.58 | 44.34 |
| LQ14d-3 | 70769220 | 69746314 | 10.46 | 0.01 | 98.65 | 95.8 | 44.26 |
| LQ21d-1 | 60439784 | 59626922 | 8.94 | 0.01 | 98.63 | 95.66 | 43.83 |
| LQ21d-2 | 62518270 | 61669262 | 9.25 | 0.01 | 98.55 | 95.49 | 43.77 |
| LQ21d-3 | 69078774 | 68332962 | 10.25 | 0.01 | 98.61 | 95.67 | 43.74 |
| LQ28d-1 | 63109046 | 62079884 | 9.31 | 0.01 | 98.84 | 96.38 | 44.02 |
| LQ28d-2 | 63061572 | 62007284 | 9.3 | 0.01 | 98.7 | 95.92 | 43.76 |
| LQ28d-3 | 68279850 | 67448908 | 10.12 | 0.01 | 98.77 | 96.16 | 43.61 |
| LQ35d-1 | 48852644 | 48224872 | 7.23 | 0.01 | 98.57 | 95.55 | 43.77 |
| LQ35d-2 | 69752626 | 68887248 | 10.33 | 0.01 | 98.56 | 95.5 | 43.7 |
| LQ35d-3 | 68245018 | 67348046 | 10.1 | 0.01 | 98.59 | 95.61 | 43.72 |
| ZF7d-1 | 68859162 | 67972592 | 10.2 | 0.01 | 98.6 | 95.66 | 44.19 |
| ZF7d-2 | 63558508 | 62570146 | 9.39 | 0.01 | 98.62 | 95.68 | 44.02 |
| ZF7d-3 | 69994098 | 69261254 | 10.39 | 0.01 | 98.67 | 95.85 | 44.17 |
| ZF14d-1 | 62259850 | 61445744 | 9.22 | 0.01 | 98.54 | 95.39 | 43.99 |
| ZF14d-2 | 66097008 | 65384242 | 9.81 | 0.01 | 98.57 | 95.53 | 44.24 |
| ZF14d-3 | 68632198 | 67860154 | 10.18 | 0.01 | 98.65 | 95.77 | 44.18 |
| ZF21d-1 | 72105924 | 70696092 | 10.6 | 0.01 | 98.62 | 95.71 | 44.26 |
| ZF21d-2 | 61631024 | 60828912 | 9.12 | 0.01 | 98.84 | 96.29 | 43.87 |
| ZF21d-3 | 78103554 | 76974614 | 11.55 | 0.01 | 98.84 | 96.37 | 44.27 |
| ZF28d-1 | 49868264 | 48952602 | 7.34 | 0.01 | 98.73 | 96.03 | 43.87 |
| ZF28d-2 | 66729876 | 65787564 | 9.87 | 0.01 | 98.7 | 95.91 | 43.68 |
| ZF28d-3 | 68592646 | 67446406 | 10.12 | 0.01 | 98.68 | 95.88 | 43.85 |
| ZF35d-1 | 82514804 | 81546396 | 12.23 | 0.01 | 98.66 | 95.85 | 44.06 |
| ZF35d-2 | 54745982 | 54065498 | 8.11 | 0.01 | 98.72 | 95.93 | 44.19 |
| ZF35d-3 | 62070910 | 61319220 | 9.2 | 0.01 | 98.69 | 95.92 | 44.29 |

**Note:** "Sample" column indicates the sample name; "Raw reads" column displays the raw read counts; "Clean reads" column shows the cleaned read counts after quality control, which are used for subsequent data analysis; "Clean base" column displays the number of bases after quality control; "Error Rate" column shows the error rate of the reads; "Q20 (%)" column displays the proportion of bases in the original sequence with a quality score> 20 (error rate <0.1%) relative to the total number of bases; "Q30 (%)" column displays the proportion of bases in the original sequence with a quality score> 30 (error rate <0.1%) relative to the total number of bases; "GC (%)" column shows the proportion of G and C bases among the cleaned reads relative to the four base types.

**Table S3.** Statistical analysis of clean reads alignment to reference genome

| Sample | Total Reads | Reads Mapped | Mapped ratio(%) | Unique mapped（%） |
|---|---|---|---|---|
| LQ14d-1 | 66079704 | 59586116 | 90.17% | 86.50% |
| LQ14d-2 | 64886704 | 58476159 | 90.12% | 86.41% |
| LQ14d-3 | 69746314 | 62856104 | 90.12% | 86.44% |
| LQ21d-1 | 59626922 | 53898410 | 90.39% | 86.80% |
| LQ21d-2 | 61669262 | 55643339 | 90.23% | 86.71% |

| | | | | |
|---|---|---|---|---|
| LQ21d-3 | 68332962 | 61677859 | 90.26% | 86.68% |
| LQ28d-1 | 62079884 | 55968091 | 90.15% | 86.33% |
| LQ28d-2 | 62007284 | 55846131 | 90.06% | 86.41% |
| LQ28d-3 | 67448908 | 60705515 | 90.00% | 86.29% |
| LQ35d-1 | 48224872 | 43216306 | 89.61% | 86.27% |
| LQ35d-2 | 68887248 | 61650941 | 89.50% | 86.02% |
| LQ35d-3 | 67348046 | 60098961 | 89.24% | 85.79% |
| LQ7d-1 | 52598542 | 47407093 | 90.13% | 86.67% |
| LQ7d-2 | 74403658 | 67141324 | 90.24% | 86.74% |
| LQ7d-3 | 75190950 | 67852981 | 90.24% | 86.64% |
| YQ14d-1 | 48382002 | 43429690 | 89.76% | 86.40% |
| YQ14d-2 | 72746830 | 65355785 | 89.84% | 86.41% |
| YQ14d-3 | 69885270 | 62821948 | 89.89% | 86.42% |
| YQ21d-1 | 63780888 | 57575661 | 90.27% | 86.81% |
| YQ21d-2 | 64242790 | 57979306 | 90.25% | 86.84% |
| YQ21d-3 | 69249104 | 62490989 | 90.24% | 86.86% |
| YQ28d-1 | 58218162 | 52352411 | 89.92% | 86.56% |
| YQ28d-2 | 55829626 | 50137106 | 89.80% | 86.49% |
| YQ28d-3 | 72519446 | 65208627 | 89.92% | 86.57% |
| YQ35d-1 | 69978808 | 62834839 | 89.79% | 86.48% |
| YQ35d-2 | 58479320 | 52591611 | 89.93% | 86.68% |
| YQ35d-3 | 78730958 | 70777384 | 89.90% | 86.50% |
| YQ7d-1 | 67147238 | 60498820 | 90.10% | 86.63% |
| YQ7d-2 | 65600492 | 59208067 | 90.26% | 86.68% |
| YQ7d-3 | 77273600 | 69612447 | 90.09% | 86.58% |
| ZF14d-1 | 61445744 | 55454954 | 90.25% | 86.82% |
| ZF14d-2 | 65384242 | 59002311 | 90.24% | 86.79% |
| ZF14d-3 | 67860154 | 61335437 | 90.39% | 86.94% |
| ZF21d-1 | 70696092 | 63887638 | 90.37% | 86.87% |
| ZF21d-2 | 60828912 | 55074926 | 90.54% | 87.15% |
| ZF21d-3 | 76974614 | 69681988 | 90.53% | 87.01% |
| ZF28d-1 | 48952602 | 44165233 | 90.22% | 86.95% |
| ZF28d-2 | 65787564 | 59511857 | 90.46% | 87.11% |
| ZF28d-3 | 67446406 | 60872113 | 90.25% | 86.79% |
| ZF35d-1 | 81546396 | 73577646 | 90.23% | 86.56% |
| ZF35d-2 | 54065498 | 48861629 | 90.37% | 86.80% |
| ZF35d-3 | 61319220 | 55336756 | 90.24% | 86.50% |
| ZF7d-1 | 67972592 | 61317138 | 90.21% | 86.79% |
| ZF7d-2 | 62570146 | 56507080 | 90.31% | 87.01% |
| ZF7d-3 | 69261254 | 62563362 | 90.33% | 87.03% |

**Note:** "Sample" column indicates the sample name; "Total Reads" column shows the total number of clean reads after quality control; "Reads Mapped" column shows the number of reads that were mapped to the reference genome; "Unique mapped" column shows the number of reads that were uniquely mapped to the reference genome.

**Table S4.** Statistical Table of Differentially Expressed Genes

| DEG Comparison | DEG Number | up-regulated | down-regulated |
|---|---|---|---|
| LQ14d vs LQ7d | 1764 | 1401 | 363 |
| LQ21d vs LQ7d | 8197 | 4759 | 3438 |
| LQ28d vs LQ7d | 6908 | 4124 | 2784 |
| LQ35d vs LQ7d | 5618 | 3397 | 2221 |
| YQ14d vs YQ7d | 3633 | 2528 | 1105 |
| YQ21d vs YQ7d | 2803 | 2002 | 801 |
| YQ28d vs YQ7d | 4338 | 3103 | 1235 |
| YQ35d vs YQ7d | 6246 | 3771 | 2475 |
| ZF14d vs ZF7d | 1169 | 446 | 723 |
| ZF21d vs ZF7d | 4545 | 2501 | 2044 |
| ZF28d vs ZF7d | 4019 | 2615 | 1404 |
| ZF35d vs ZF7d | 6882 | 3698 | 3184 |

**Note:** "DEG Comparison" indicates the groups being compared; "DEG Number" indicates the total number of differentially expressed genes; "up-regulated" indicates the number of upregulated differential genes; "down-regulated" indicates the number of downregulated differential genes.

**Table S5.** *CmMADS* gene family members and physicochemical properties of their encoded proteins

| Gene Name | Sequence ID | Gene ID | Length （aa） | Mw （kDa） | pI | Instability Index | Sub-cellular Localization |
|---|---|---|---|---|---|---|---|
| *CmMADS1* | GWHPANWH003070 | *EVM0011652* | 391 | 44.593 | 5.09 | 67.73 | Nucleus |
| *CmMADS2* | GWHPANWH003319 | *EVM0001334* | 244 | 27.931 | 9.1 | 40.47 | Nucleus |
| *CmMADS3* | GWHPANWH003321 | *EVM0031558* | 243 | 27.796 | 9.03 | 62.9 | Nucleus |
| *CmMADS4* | GWHPANWH004169 | *EVM0008719* | 217 | 25.276 | 7.62 | 65.73 | Nucleus |
| *CmMADS5* | GWHPANWH005538 | *EVM0029613* | 225 | 25.924 | 9.33 | 42.95 | Nucleus |
| *CmMADS6* | GWHPANWH005955 | *EVM0005347* | 268 | 30.427 | 6.08 | 58.59 | Nucleus |
| *CmMADS7* | GWHPANWH007290 | *EVM0028266* | 189 | 21.95 | 9.35 | 48.08 | Nucleus |
| *CmMADS8* | GWHPANWH007806 | *EVM0030064* | 242 | 27.894 | 9.59 | 53.01 | Nucleus |
| *CmMADS9* | GWHPANWH007914 | *EVM0033476* | 214 | 24.095 | 8.94 | 54.26 | Nucleus |
| *CmMADS10* | GWHPANWH007915 | *EVM0017991* | 217 | 24.135 | 8.76 | 50.97 | Nucleus |
| *CmMADS11* | GWHPANWH007916 | *EVM0003542* | 187 | 20.85 | 9.71 | 41.01 | Nucleus |
| *CmMADS12* | GWHPANWH010305 | *EVM0004314* | 241 | 27.087 | 9.62 | 36.85 | Nucleus |
| *CmMADS13* | GWHPANWH010307 | *EVM0007003* | 219 | 24.761 | 9.63 | 42.7 | Nucleus |
| *CmMADS14* | GWHPANWH010308 | *EVM0023289* | 209 | 23.541 | 8.3 | 36.43 | Nucleus |
| *CmMADS15* | GWHPANWH010436 | *EVM0025277* | 243 | 27.873 | 9.03 | 44.77 | Nucleus |
| *CmMADS16* | GWHPANWH010488 | *EVM0018777* | 227 | 25.841 | 5.63 | 55.77 | Nucleus |
| *CmMADS17* | GWHPANWH010489 | *EVM0032165* | 217 | 24.9 | 5.63 | 60.78 | Nucleus |
| *CmMADS18* | GWHPANWH010490 | *EVM0015139* | 217 | 24.843 | 5.8 | 61.14 | Nucleus |
| *CmMADS19* | GWHPANWH010493 | *EVM0011070* | 254 | 28.855 | 7.05 | 57.76 | Nucleus |
| *CmMADS20* | GWHPANWH010497 | *EVM0019787* | 220 | 25.283 | 6.25 | 56.98 | Nucleus |

| | | | | | | | |
|---|---|---|---|---|---|---|---|
| *CmMADS21* | GWHPANWH010823 | *EVM0005110* | 248 | 28.928 | 6.52 | 56.16 | Nucleus |
| *CmMADS22* | GWHPANWH012627 | *EVM0012385* | 202 | 23.332 | 9.61 | 57.51 | Nucleus |
| *CmMADS23* | GWHPANWH014553 | *EVM0001625* | 217 | 25.235 | 7.64 | 60.9 | Nucleus |
| *CmMADS24* | GWHPANWH014810 | *EVM0003703* | 317 | 36.822 | 9.02 | 47.56 | Nucleus |
| *CmMADS25* | GWHPANWH014895 | *EVM0002609* | 209 | 23.861 | 9.31 | 50.92 | Nucleus |
| *CmMADS26* | GWHPANWH015184 | *EVM0021210* | 149 | 17.052 | 9.68 | 52.62 | Nucleus |
| *CmMADS27* | GWHPANWH015254 | *EVM0009567* | 253 | 29.029 | 8.97 | 52.19 | Nucleus |
| *CmMADS28* | GWHPANWH015896 | *EVM0006812* | 206 | 23.434 | 9.61 | 43.64 | Nucleus |
| *CmMADS29* | GWHPANWH016150 | *EVM0021399* | 256 | 29.361 | 9.32 | 44.75 | Nucleus |
| *CmMADS30* | GWHPANWH017130 | *EVM0003577* | 250 | 28.147 | 7.69 | 35.73 | Nucleus |
| *CmMADS31* | GWHPANWH017143 | *EVM0017452* | 236 | 26.512 | 8.62 | 38.9 | Nucleus |
| *CmMADS32* | GWHPANWH017812 | *EVM0011784* | 240 | 27.562 | 8.86 | 48.27 | Nucleus |
| *CmMADS33* | GWHPANWH017813 | *EVM0006633* | 224 | 25.692 | 9.56 | 51.46 | Nucleus |
| *CmMADS34* | GWHPANWH018581 | *EVM0031920* | 388 | 44.08 | 6.84 | 51.36 | Nucleus |
| *CmMADS35* | GWHPANWH018742 | *EVM0001268* | 242 | 27.672 | 8.19 | 49.41 | Nucleus |
| *CmMADS36* | GWHPANWH018743 | *EVM0001793* | 246 | 28.511 | 8.71 | 56.85 | Nucleus |
| *CmMADS37* | GWHPANWH019201 | *EVM0000946* | 250 | 28.517 | 9.01 | 55.84 | Nucleus |
| *CmMADS38* | GWHPANWH020503 | *EVM0030737* | 256 | 28.999 | 6.55 | 55.61 | Nucleus |
| *CmMADS39* | GWHPANWH024641 | *EVM0027757* | 243 | 28.662 | 4.82 | 65.37 | Nucleus |
| *CmMADS40* | GWHPANWH025552 | *EVM0011483* | 281 | 32.404 | 8.58 | 58.95 | Nucleus |
| *CmMADS41* | GWHPANWH025553 | *EVM0002586* | 244 | 27.676 | 8.88 | 42.62 | Nucleus |
| *CmMADS42* | GWHPANWH025717 | *EVM0023646* | 364 | 41.481 | 6.23 | 45.28 | Nucleus |
| *CmMADS43* | GWHPANWH027065 | *EVM0019430* | 233 | 26.955 | 9.48 | 50.18 | Nucleus |
| *CmMADS44* | GWHPANWH027824 | *EVM0007180* | 222 | 25.377 | 9.31 | 55.3 | Nucleus |
| *CmMADS45* | GWHPANWH027826 | *EVM0006578* | 421 | 47.595 | 8.84 | 38.57 | Nucleus |
| *CmMADS46* | GWHPANWH027901 | *EVM0028000* | 225 | 26.14 | 9.35 | 41.01 | Nucleus |
| *CmMADS47* | GWHPANWH027987 | *EVM0026273* | 227 | 25.878 | 6.27 | 58.85 | Nucleus |
| *CmMADS48* | GWHPANWH028041 | *EVM0023922* | 233 | 26.984 | 8.73 | 51.42 | Nucleus |
| *CmMADS49* | GWHPANWH028089 | *EVM0021973* | 199 | 21.581 | 6.17 | 36.64 | Nucleus |
| *CmMADS50* | GWHPANWH030280 | *EVM0025593* | 406 | 43.669 | 7.75 | 38.3 | Nucleus |
| *CmMADS51* | GWHPANWH031147 | *EVM0026806* | 236 | 26.755 | 6.35 | 44.53 | Nucleus |
| *CmMADS52* | GWHPANWH031155 | *EVM0017842* | 246 | 27.85 | 6.42 | 45.26 | Nucleus |
| *CmMADS53* | GWHPANWH031428 | *EVM0024725* | 211 | 24.06 | 8.59 | 53.72 | Nucleus |
| *CmMADS54* | GWHPANWH032071 | *EVM0029707* | 241 | 27.773 | 8.37 | 50.09 | Nucleus |
| *CmMADS55* | GWHPANWH032159 | *EVM0012233* | 221 | 25.359 | 9.32 | 45.51 | Nucleus |
| *CmMADS56* | GWHPANWH032665 | *EVM0003992* | 209 | 24.489 | 6.62 | 61.86 | Nucleus |
| *CmMADS57* | GWHPANWH033056 | *EVM0024674* | 337 | 38.97 | 8.68 | 54.84 | Nucleus |
| *CmMADS58* | GWHPANWH000084 | *EVM0008980* | 164 | 19.063 | 6.24 | 61.04 | Nucleus |
| *CmMADS59* | GWHPANWH000760 | *EVM0008265* | 204 | 23.401 | 10.0 | 68.31 | Nucleus |
| *CmMADS60* | GWHPANWH001084 | *EVM0001009* | 164 | 19.063 | 6.24 | 61.04 | Nucleus |

**Note:** "Gene Name" indicates the gene name of the chestnut MADS-box family; "Sequence ID" indicates the protein sequence identifier in the chestnut genome; "Gene ID" indicates the gene identifier in the chestnut genome; "Length (aa)" specifies the number of amino acids; "Mw (kDa)" represents the molecular weight; "pI" stands for the

isoelectric point; "Sub-cellular Localization" indicates the predicted subcellular localization.

**Table S6.** Ka, Ks and Ka/Ks of duplication pairs of MADS-box gene family in *C.mollissima*

| Duplicated gene pairs | Non synonymous(Ka) | Synonymous (Ks) | Ka/Ks | Duplicated type |
|---|---|---|---|---|
| *CmMADS2/CmMADS35* | 0.233548553 | 1.4713582 | 0.158729909 | segmental |
| *CmMADS2/CmMADS41* | 0.15890355 | 0.947007312 | 0.167795484 | segmental |
| *CmMADS3/CmMADS40* | 0.203482067 | 1.903895806 | 0.106876682 | segmental |
| *CmMADS5/CmMADS46* | 0.334685356 | 1.388251727 | 0.241084055 | segmental |
| *CmMADS55/CmMADS18* | 0.363497959 | 1.217415039 | 0.298581788 | segmental |
| *CmMADS54/CmMADS37* | 0.326643751 | 1.018096485 | 0.320837716 | segmental |
| *CmMADS55/CmMADS47* | 0.297178295 | 1.991087379 | 0.149254271 | segmental |
| *CmMADS13/CmMADS49* | 0.653412124 | 3.017959657 | 0.216507905 | segmental |
| *CmMADS17/CmMADS47* | 0.225490781 | 1.329541697 | 0.169600383 | segmental |
| *CmMADS17/CmMADS18* | 0.01783734 | 0.09881442 | 0.180513527 | tandem |
| *CmMADS46/CmMADS47* | 0.588484347 | 2.66530283 | 0.220794553 | tandem |

**Note**：Ka/Ks < 1 indicates purifying selection

**Table S7.** Primers used for transcriptome sequencing verification and primer efficiency

| Gene Name | Forward primer (5' to 3') | Reverse primer (5' to 3') | Primer efficiency |
|---|---|---|---|
| *CmMADS8* | ACCCGAACCAATCCATGTCG | GCAACCTCAGCATCACACAG | 96.96% |
| *CmMADS16* | GGCAAGGCAGGTGACATTCT | GCATACTCGAGCTGGCGTAT | 97.58% |
| *CmMADS36* | TTCCAAAAGGAGAGGTGGGC | CCATGCAAGAATCTGTGGCG | 97.49% |
| *CmMADS56* | TGAGCTCAGGCACTTGAAGG | GTTGGTGCAGAACGAGGGTA | 95.71% |
| *CmActin* | TTGACTATGAGCAGGAACTT | TTGTAGGTGGTCTCGTGAAT | 99.19% |
